# The COVID-19 Infodemic on Twitter: A Space and Time Topic Analysis of the Brazilian Immunization Program and Public Trust

**DOI:** 10.3390/tropicalmed7120425

**Published:** 2022-12-09

**Authors:** Victor Diogho Heuer de Carvalho, Thyago Celso Cavalcante Nepomuceno, Thiago Poleto, Ana Paula Cabral Seixas Costa

**Affiliations:** 1 Eixo das Tecnologias, Campus do Sertão, Universidade Federal de Alagoas, Delmiro Gouveia 57480-000, Brazil; 2 Núcleo de Tecnologia, Centro Acadêmico do Agreste, Universidade Federal de Pernambuco, Caruaru 55014-900, Brazil; 3 Departamento de Administração, Instituto de Ciências Sociais Aplicadas, Universidade Federal do Pará, Belém 66075-110, Brazil; 4 Departamento de Engenharia de Produção, Centro de Tecnologia e Geociências, Universidade Federal de Pernambuco, Recife 50740-550, Brazil

**Keywords:** COVID-19, infodemic, misinformation, Twitter, topic analysis, immunization program, public trust, Brazil

## Abstract

The context of the COVID-19 pandemic has brought to light the infodemic phenomenon and the problem of misinformation. Agencies involved in managing COVID-19 immunization programs are also looking for ways to combat this problem, demanding analytical tools specialized in identifying patterns of misinformation and understanding how they have evolved in time and space to demonstrate their effects on public trust. The aim of this article is to present the results of a study applying topic analysis in space and time with respect to public opinion on the Brazilian COVID-19 immunization program. The analytical process involves applying topic discovery to tweets with geoinformation extracted from the COVID-19 vaccination theme. After extracting the topics, they were submitted to manual annotation, whereby the polarity labels pro, anti, and neutral were applied based on the support and trust in the COVID-19 vaccination. A space and time analysis was carried out using the topic and polarity distributions, making it possible to understand moments during which the most significant quantities of posts occurred and the cities that generated the most tweets. The analytical process describes a framework capable of meeting the needs of agencies for tools, providing indications of how misinformation has evolved and where its dissemination focuses, in addition to defining the granularity of this information according to what managers define as adequate. The following research outcomes can be highlighted. (1) We identified a specific date containing a peak that stands out among the other dates, indicating an event that mobilized public opinion about COVID-19 vaccination. (2) We extracted 23 topics, enabling the manual polarity annotation of each topic and an understanding of which polarities were associated with tweets. (3) Based on the association between polarities, topics, and tweets, it was possible to identify the Brazilian cities that produced the majority of tweets for each polarity and the amount distribution of tweets relative to cities populations.

## 1. Introduction

The pandemic has significantly impacted people’s daily lives worldwide, affecting work, education, and social interactions and leading to the enforcement of distancing measures worldwide [1,2,3]. Physical distancing made people interact predominantly through virtual and online platforms such as social networks, video-conference software, and instant messenger applications [4]. In addition to adopting home-based work [5], these interactions generated large volumes of data that can be retrieved for various types of analysis [6,7], supporting strategies to mitigate the COVID-19 outbreak, from early detections to monitoring infodemic phenomena based on people’s opinions and emotions, and the misinformation spread through the social web [8].

The opinions and sentiments expressed by people about aspects of the pandemic have triggered worldwide research seeking to analyze the associated patterns and generate informational inputs to support actions in favor of combating the social effects of COVID-19 [9]. Among the themes studied by researchers worldwide based on opinions and sentiment analysis, the construction of corpora about the pandemic can be considered a starting point for any related analysis (see, for instance, [10,11,12,13,14]).

In this context, Cotfas et al. [15] analyzed public opinion about COVID-19 vaccination in the United Kingdom. Topics were extracted using latent Dirichlet allocation (LDA) to support the analysis of the opinions. The authors suggested that their findings can be used to combat public hesitation with respect to vaccines, as information about the causes of such hesitation can be extracted through topics.

Abdulaziz et al. [10] also focused on sentiment analysis of COVID-19, using LDA to extract topics from Twitter texts and the Valence Aware Dictionary and Sentiment Reasoner (VADER) lexicon model to classify text sentiments. Using these techniques, they identified trending topics during the pandemic, including “drugs research”, “news”, “losses”, ‘economy”, “lockdown”, “updated cases”, “school closures”, and “rules”, separating the topics according to positive, neutral, and negative labels.

Melton et al. [16] applied public sentiment analysis using information shared in Reddit communities about COVID-19 vaccines, using the LDA to model and extract topics, classifying them according to their sentiment polarity (“positive”, “negative”, or “neutral”). The study results showed that Reddit communities expressed more positive than negative opinions about vaccine-related content, maintaining this tendency over time.

Shi et al. [17] developed a study on emotional analysis based on public opinion among the Chinese population, using posts from the Weibo microblog platform. In their analytical process, they applied LDA for topic discovery and text emotion extraction using a pre-existing emotional ontology vocabulary and analyzed the evolution of emotional timelines for Chinese cities, in addition to identifying the most discussed topics during the first wave of the COVID-19 pandemic.

In a study with a medical infoveillance orientation, Mackey et al. [18] applied topic modeling using the Biterm Topic Model (BTM) to detect and characterize conversations among Twitter users that were COVID-19-related. The authors reported that tweets could be separated into five kinds: those related to reports of symptoms, those involving symptom reporting concurrent with lack of testing, those containing discussions about recovery, those containing discussions about test confirmations reporting a negative COVID-19 diagnosis, and those recalling symptoms and questioning whether they had already contracted the disease.

Daradkeh [19] presented an analytical framework for topic modeling COVID-19 misinformation on social media using LDA, sentiment analysis, and VADER. The topic model consisted of vaccination policy and strategy, society and community, organizations and institutions, family and friends, vaccination behavior, and vaccination experience. Sentiment analysis separated tweets into four classes: misinformation combined with positive sentiment, misinformation combined with negative sentiment, non-misinformation with positive sentiment, and non-misinformation with negative sentiment, enabling the generation of a sentiment trend timeline over six months.

Wang et al. [20] analyzed topic and feature distribution relative to the need for public information on COVID-19 using texts extracted from a Chinese online question-and-answer community. Their methodology applied LDA and the *k*-means clustering algorithm, obtaining as results keywords related to the public information about vaccination, a co-keywords network, and an “information needs framework” based on the results of both methods, elucidating, for instance, the information needs of various social groups.

Zhu et al. [21] developed a public opinion analysis framework based on LDA for topic modeling and Bidirectional Encoder Representations from Transformers (BERT) with Functional Data Analysis (FDA) for sentiment classification using data collected from Weibo platform in China. A remarkable result of their research was the distinction between positive and negative posts, revealing differences in the topics with vaccine-support and vaccine-hesitant orientations.

Xie et al. [22] reviewed the Chinese government’s information about COVID-19 to identify scientific communication topics. They applied LDA, correlation analysis, and analysis of variance (ANOVA) on a collection of news briefings composing a science-related corpus. They found topics related to communication about (1) prevention, control, epidemiological investigation, and personal health according to the popularization of scientific knowledge models; (2) research on Chinese medicine, vaccines, and medical resources according to the public understanding of scientific knowledge; and (3) citizen, community, and enterprise participation in scientific knowledge co-production.

Another interesting study related to the pandemic was developed by Zhou et al. [23] in China to assess the spatio-temporal characteristics of public sentiments about COVID-19 in small cities, supporting public agencies to decide on anti-pandemic measures. They applied a dictionary-based sentiment analysis, local Moran’s index, and kernel-density analysis to support analyzing non-linear evolution and clustering characteristics of public sentiment in space and in time.

The vaccination campaign against COVID-19 in Brazil was marked by the division of public opinion, with discussions and confrontations between pro- and anti-vaccination groups on social networks and the notable dissemination of misinformation about vaccines [24,25]. Political interference in support of both movements in Brazil took this discussion to a different level, leading to an investigation by the Brazilian Senate into potential crimes committed throughout the entire process of fighting COVID-19, from sanitary and isolation measures to the vaccines’ purchasing [26] and even considering politicians’ negationism about the pandemic [27].

The dissemination of misinformation about COVID-19 vaccination in Brazil and the world brought to light the need for immunization program management agencies to properly analyze the infodemic phenomenon [27]. It includes analytical tools able to demonstrate how misinformation evolves in time and space, ensuring the understanding of where and when it emerged, understanding the effects on the population, and making it possible to replace “wrong” with “right” information [24,28,29].

Some recent studies have indicated paths to be followed to address this need. We highlight those that are most associated with the research we carry out: applying methods to discover patterns in comments and opinions indicating the commenter’s behavior [30]; systematizing the assessment of underlying exogenous variables related to people’s belief in misinformation [31]; building datasets with significant amounts of texts to be used by analytical tools, providing reliable results, considering texts with geographic information, and obtaining them from multiple channels to extend the studies validity [10,25,32].

The present article reports a new study expanding the findings of de Carvalho et al. [33], who applied temporal opinion analysis to tweets about COVID-19 vaccination in Brazil. The main objective is to present the results of a spatio-temporal analysis of a set of geocoded tweets, demonstrating which topics were discussed between 2020 and 2021 through an interface that considers the distribution of posts over time and the Brazilian geographic space.

This study attempts to address the needs identified above by applying a methodological approach that public agencies managing the COVID-19 immunization program can use to combat the spread of misinformation, considering natural language processing tools, text mining, and machine learning to provide structured and helpful information.

Four research questions can be defined regarding our research problem:

(1) What topics were discussed during 2020 and 2021 on COVID-19 immunization in Brazil?
(2) What can be understood/interpreted about the extracted topics?
(3) How did topics evolve over time?
(4) How are they distributed in Brazilian territory, and which places have generated the most tweets?

Among the research findings, we highlight the most interesting: (1) the highest peak in the number of tweets for most of the topics and all polarities on 17 January 2021, the date of the first COVID-19 vaccine applied in Brazil; (2) twenty-three topics were extracted and, after manual annotation, fifteen of them received the pro-vaccination label, five the neutral label, and three the anti-vaccination label, indicating a predominance of topics supporting vaccination in Brazil within the period studied, according to the annotators’ judgments; (3) through the association *polarity → topic → tweet*, it was possible to identify the Brazilian cities that produced the most tweets according to each polarity, and the distribution of the number of tweets according to the cities’ population.

## 2. Methods

The methodological approach applied in our research was based on a social web mining framework [34] with the following main elements:

(1) Texts collection from Twitter
(2) Texts preprocessing
(3) Feature selection
(4) Topic modeling and discovery
(5) Spatio-temporal topic analysis

**Figure 1:**
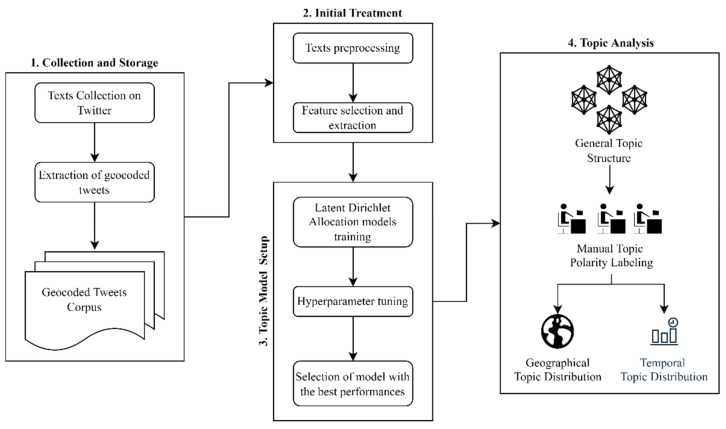
Workflow for the spatio-temporal topic analysis.

Figure 1 describes the workflow of this framework, divided into four parts, starting with the data acquisition and ending with the exploration of topic distribution in space and time.

### 2.1. Texts Collection and Storage

The texts were collected using Twitter API 2.0 through a scraping script developed in Python, applying functions from the “requests” library. Larger corpora were constructed, as reported in de Carvalho et al. [33], but a subset containing only geocoded tweets was extracted, composing a georeferenced corpus. Table S1 in the Supplementary Materials presents the parameters used in the scraping script. The blocks of keywords applied for scraping are based on the studies by de Oliveira et al. [35] and de Carvalho et al. [33,36].

In the final steps of the scraping process, there is a preliminary treatment to exclude fields from tweets unrelated to this search. The final georeferenced corpus was stored in a comma-separated values (csv) file with the following fields:

(1) Tweet text (*text* field)
(2) Place name (*place_name* field)
(3) Country name (*country* field)
(4) Geographic coordinates (*point* field)
(5) Creation date (*created_at* field)

The geographic coordinates field consists of *latitude* and *longitude*, and it was later decomposed into specific vectors for each part of the coordinate. The dates were transformed into a readable format by the temporal analysis scripts.

### 2.2. Initial Treatment

A set of preprocessing functions was applied to minimize textual noise effects: case folding; duplicities exclusion; tasks for eliminating punctuation, emojis/emoticons, numbers, stop words, links/URLs; and tokenization. The preprocessing was developed through another Python script using mainly the Spacy library [37], which implements all the listed functions, and the Pandas library [38] for data frame manipulation and to convert data frames to the final storage file format (csv).

After preprocessing, the texts were vectorized using simple term counting (defining the frequency of the term) and the term frequency-inverse document frequency (TF-IDF). Both vectorization processes were applied using the respective functions available through the Scikit-Learn framework [39].

Each vectorization method received the lemmatized tokens extracted from the tweets. So, for the subsequent topic modeling and analysis phases, the words (treated as uni-grams) were the selected features.

### 2.3. Topic Model Setup

Topic modeling methods consist of converting numeric (document × term) vectors representing documents to (topic × term) and (document × topic) vectors [40]. The topic modeling applied in our study was based on the Latent Dirichlet Allocation (LDA), a hierarchical probabilistic model to decompose a collection of documents in topics based on probability distributions of vocabularies [41].

The model was proposed and described by Blei et al. [42] and can be represented by the generative process in Algorithm 1, where *N* denotes a sequence of words *w* = (*w*_1_, *w*_2_, …, *w_n_*), *θ* is a *k*-dimensional Dirichlet random variable that can assume values in the (*k*—1)-*simplex* vector. In our study, we used Scikit-Learn’s LDA implementation considering the online variational Bayes method [43], using mini-batches of training data to update the number of components incrementally.
**Algorithm 1** Latent Dirichlet Allocation Generative Process1.For each document *w* in a corpus *D*:1.1Choose
$N\;\sim \;Poisson\left(\xi \right)$
1.2Choose $\theta \;\sim \;Dirichlet\left(\alpha \right)$1.3For each of the *N* words *w_n_*:1.3.1Choose a topic $z_{n}\;\sim \;Multinomial\left(\theta \right)$1.3.2Choose a word *w_n_* from $p(w_{n}|z_{n},\;\beta )$, a multinomial probability related to the topic *z_n_*

A hyperparameter tuning using Scikit-Learn’s Grid Search function was performed, considering learning decays of 0.5, 0.7, and 0.9 for between 3 and 30 topics. The learning decays are parameters that control the learning rate when using the LDA online variational Bayes method and can vary from 0.3 to 1.0.

The Grid Search can deliver test scores such as the model perplexity and the log-likelihood ($\log p\left(w_{d}\right))$ for hyperparameter combinations. We can interpret the perplexity as a measure of the possibilities of the collection of unseen words of a text belonging to a topic, and the lower it is, the better the LDA model’s performance [44]; the higher the log-likelihood, the better the model [42]. Scikit-Learn’s perplexity function calculates the related score as $exp(-1\times \log p\left(w_{d}\right))$.

### 2.4. Topic Analysis

The previous phase outputs enable the topic analysis. For the overall topic visualization, we used (1) intertopic distance mapping, (2) the most salient terms evidencing, and (3) topic segregation. For the intertopic distance mapping and the most salient terms, we used a Python library called pyLDAvis [45,46], automatically creating dashboards for topic visualization.

In the intertopic distance map, the extracted topics are represented by circles, and their area corresponds to each topic’s term prevalence. The center of each topic is defined by calculating the distance between the topics, using a multidimensional scaling process to represent the distances on a two-dimensional plane [47]. The process to obtain the most salient terms consists of applying a saliency measure to filter and rank the terms, considering Equations (2) and (3) described by Chuang et al. [48]:

(2) $$istinctiveness\left(w\right)=\underset{T}{\sum }P(T|w)\times log\frac{P(T|w)}{P\left(T\right)}$$

(3) $$saliency\left(w\right)=P\left(w\right)\times distinctiveness\left(w\right)$$

According to Chuang et al. [48], Equation (2) describes how informative the specific term *w* is for determining the generating topic *T*, versus a randomly-selected term *w’*. Equation (3) describes how to calculate the salience measure, considering that given the number of words, the list of the most probable terms contains more generic words than the list of specific terms [48].

For topic segregation, we used the *t*-distributed stochastic neighbor embedding (*t*-SNE) implemented in Scikit-Learn and the Bokeh library [49] for plotting. The *t*-SNE was designed to alleviate the crowding problem in the traditional stochastic neighbor embedding (SNE) using a symmetric version of the SNE cost function [50]. Joint probabilities *p_ij_* described by Equations (4) and (5) are defined to measure similarities between objects *x_i_* and *x_j_*, symmetrizing two conditional probabilities, where *σ_i_* represents the bandwidth of Gaussian kernels defined so that the perplexity of the conditional distribution *P_i_* is equal to a predefined perplexity *u* [50,51].

(4) $$p_{i|j}=\frac{\exp(-d{\left(x_{i},\;x_{j}\right)}^{2}/2\sigma_{i}^{2}}{\sum_{k\neq i}exp\left(-d{\left(x_{i},\;x_{k}\right)}^{2}/2\sigma_{i}^{2}\right)}$$

(5) $$p_{i|j}=\frac{p_{j|i}+p_{i|j}}{2N}$$

The final part of the methodologic workflow is the space and time topic analysis. Using the results of the previous phase, we can perform the following tasks:

(1) Manual topic polarity labeling, dedicated to annotating the polarity trend associated with each topic into three types:
  (1) Supportive topic (pro), with positive relation, denoting trust about the COVID-19 vaccination.
  (2) Negationist or unsupportive topic (anti), with negative relation, denoting mistrust about vaccination.
  (3) Neutral topic (neutral), not associated with the types above.
(2) Geographical Topic Analysis is dedicated to visualizing and understanding the topics’ distribution in a geographic area. There are two possible ways to perform this analysis [52]: (1) discovering different topics of interest that are coherent across geographic regions and (2) comparing several topics across different geographical locations.
(3) Time Topic Analysis is dedicated to visualizing and understanding the topics’ distribution over time. One example of this application can be seen in the study by Martin et al. [53].

In our research, the manual topic polarity annotation was performed by an initial group of three scholars, from different research areas, with different backgrounds: Informatics, Economics, and Life/Health Sciences. This manual labeling becomes necessary to ensure an alignment based on human knowledge and the consequent reliability of the topics concerning their positioning on the investigated theme. The use of three annotators is a way to ensure that, if there is a disagreement between the labels of two of them, the labeling of a third one can serve as a basis to define which polarity label will be defined [54]. If there is still a tie, even with the judgments of the three annotators, a fourth annotator will be invited to perform the tiebreakers only for the topics that caused indecision.

The Brazilian geographic space is considered for the analysis performed in this study, and the geographical topic analysis is aligned with the idea of discovering topics of interest across the Brazilian geographic regions. Using data collected from June 2020 to October 2021, we could perform the time topic analysis, demonstrating the evolution of the discussions. Graphs were developed using the Python Matplotlib library [55], and the geographical features were handled using the GeoPandas library [56].

## 3. Results and Discussion

The results will be presented following, divided into (1) the general findings, considering initial data about the corpus constructed for the spatio-temporal analysis; (2) the topic modeling results according to the Grid Search applied; and (3) topic analysis findings, considering the manual labeling, and the spatio-temporal analysis.

### 3.1. General Findings

**Figure 2:**
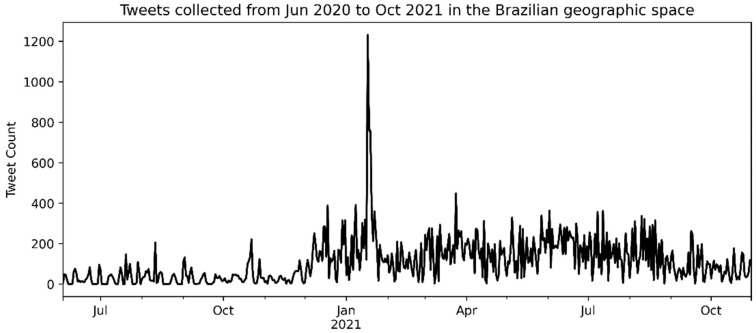
Overall tweets distribution over 17 months.

Considering the geocoded tweets extracted from one of the corpora presented by de Carvalho et al. [33], the initial corpus composition for the present study contained 62,873 tweets. We applied a cleaning process to eliminate tweets that escaped location and language filters, resulting in 55,758 tweets. Table S2 in the Supplementary Materials contains an example of georeferenced corpus content, with its first five lines. Figure 2 presents the tweets’ amounts over the seventeen months (from June 2020 to October 2021).

**Figure 3:**
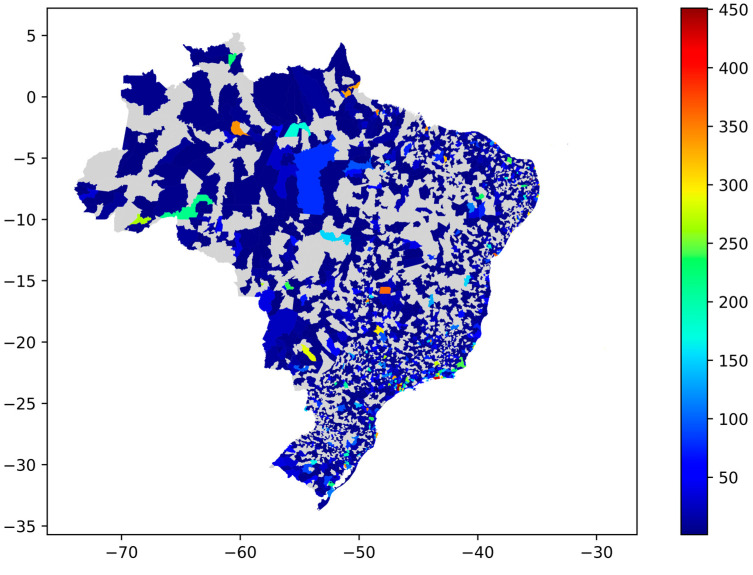
Overall tweets distribution in Brazilian territory.

The number of tweets started to increase in mid-December 2020; in addition, there was a peak with 1224 tweets on 17 January 2021, identified by analyzing the collected data. Both situations were also noted in the study by de Carvalho et al. [33]. Figure 3 presents the general distribution of tweets according to the cities that generated them in the Brazilian municipal grid.

There are twenty-five cities with at least 300 tweets registered in the corpus; among them, twenty cities are state capitals, including Brasília (the country capital), and the others are major urban centers in their respective states. Table S3 in the Supplementary Materials presents the list of these twenty-five cities with the associated number of tweets. In this map and all the others along the results, the cities are represented in the Brazilian municipal grid. In other words, for figures with maps, although we refer to the cities, the graphical representations are the municipalities.

### 3.2. Topic Modeling Results

The topic modeling process considered two word-vectors, one obtained using the count vectorizer and the other the TF-IDF vectorizer. The main results for the Grid Searches using these vectors were:

(1) For the words vector obtained with the count method: a topic model with only three components (topics), using a learning decay equals 0.7. This result is corroborated by the best log-likelihood of −840,183.6751 and perplexity of 1479.7684.
(2) For the words vector obtained with TF-IDF method, a topic model with only three components, and in this case, using a leaning decay equals 0.5. This result is corroborated by the log-likelihood of −281,057.5610 and perplexity of 4225.0856.

**Figure 4:**
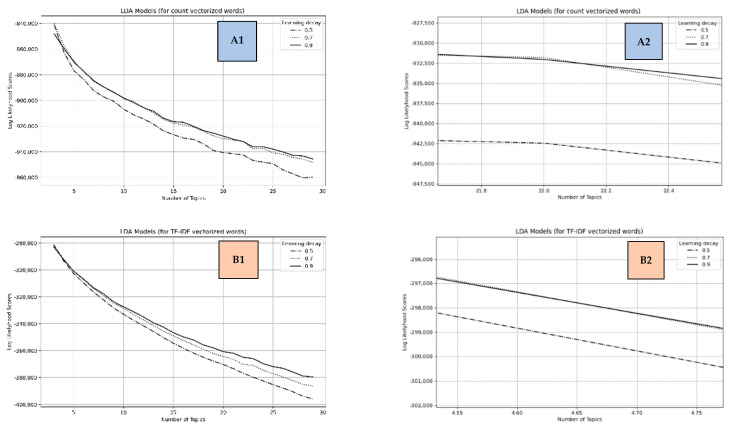
Learning decay curves for both words’ vectors: graphs (**A1**,**B1**) contain the overall curves for the traditional count (frequency), and the TF-IDF vectorized words, successively; graphs (**A2**,**B2**) present the regions where the last overcome between the curves occurred, supporting defining the best topic model.

However, the results of this test require a sensitivity analysis that can be applied using the learning decay’s performance graphs. This analysis can help define a more significant number of topics within the determined limits. Although the increase in the number of topics for each learning decay may cause a decrease in the log probability (whose values are negative) regardless of the applied vectorization, there will be a better margin of topics for analysis based on the diversification of the sub-themes involved. The graph grid in Figure 4 contains the overall performance curves for each of the three learning decays.

Graphs A1 and B1 in Figure 4 corroborate the best topic models with only three topics, but we can refine these results by looking for the graphs’ regions where the initial best-performing learning decay curves are overcome.

Learning decay curves for the words vector obtained using the count vectorizer presented the definitive overcoming of the learning decay curve of 0.7 between 22 and 22.5 for the number of topics. The curve initially containing the best result was surpassed by the learning decay curve of 0.9, as presented in Graph A2 (Figure 4). So, for this case, we can define 23 as the number of topics, rounding this number after 22.5. The learning decay curve of 0.5 in this analysis always remained below the other two.

For the learning decay curves related to the words’ vectors obtained using the TF-IDF vectorizer, there were several points where the curves of 0.5 and 0.7 crossed each other. For instance, the first time was between 3.10 and 3.15 topics, where the learning curve of 0.5 (containing the initial best model) was overcome by the curve of 0.7. However, as recorded in Graph B2 (Figure 4), the learning curve of 0.7 was overcome by 0.9 between 4.60 and 4.70 topics. After this, there was no other overcoming among the curves. It indicated a model with five topics, rounding this number of topics immediately after 4.7 and the learning decay of 0.9.

Based on this sensitivity analysis, we opted to use a model with 23 topics according to the words’ vector obtained through the count method.

### 3.3. Topic Analysis Findings

**Figure 5:**
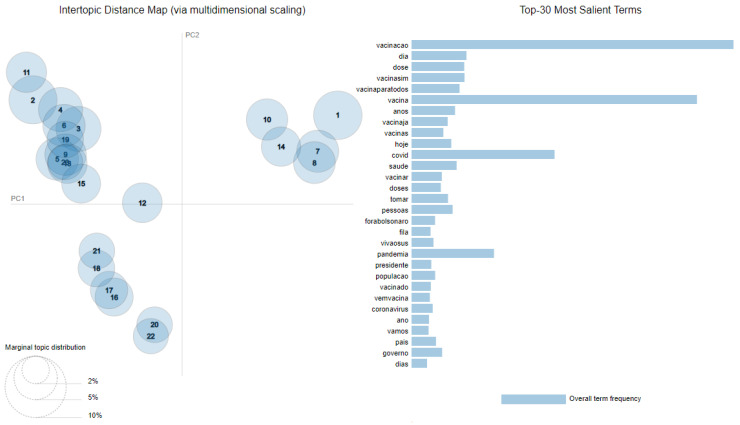
Intertopic distance map and the most salient terms found in the extraction process [45,48].

The initial output of the topic modeling and extraction is the overall topic structure. There are several forms to present this structure. Table S4 in the Supplementary Materials presents the 15 most frequent terms for each topic, and Table S5 translates these terms into English. A graphical visualization for the topics is given in Figure 5.

The graphs in Figure 5 support understanding the distribution and proximity of each topic in a two-dimensional projection and the most salient terms that can be interpreted as the most frequent words between all the topics. The thirty most salient terms, translated to English, are: vaccination, day, shot, vaccineyes, vaccineforeverybody, vaccine, years, vaccinenow, vaccines, today, covid, health, to vaccinate, shots, to take, people, outbolsonaro, queue, hailthesus, pandemic, president, population, vaccinated, comevaccine, coronavirus, year, [we] will, country, government, and days. Some of these terms, before preprocessing, contained hashtags (#):

- vaccineyes → vaccine yes
- vaccineforeverybody → vaccination for everybody
- vaccinenow → vaccine now
- outbolsonaro → out Bolsonaro (the family name of the Brazilian president at the time this study was carried out)
- hailthesus → hail the SUS (the last part is the acronym for *Sistema Unico de Saúde*, the Brazilian Public Health System)
- comevaccine → come vaccine

**Figure 6:**
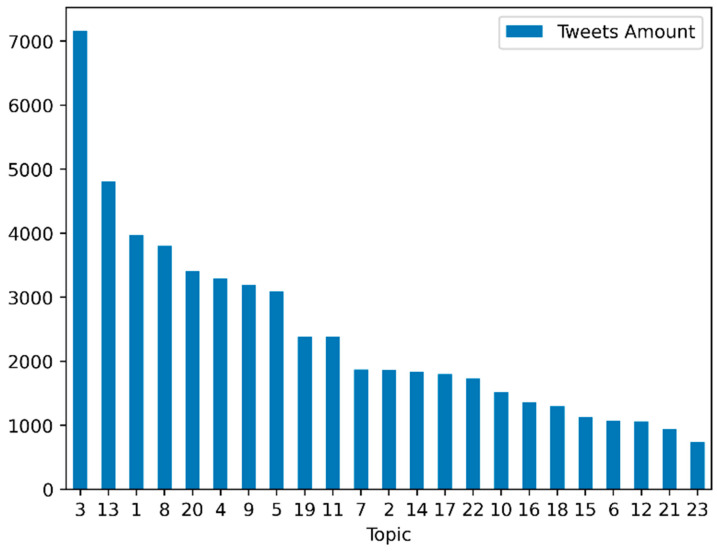
Tweets distributions among the 23 extracted topics.

See Table S6 in the Supplementary Materials for the interpretations of what each topic can mean. The number of tweets on each topic is presented in Table S7 in the Supplementary Materials. Figure 6 presents the distribution of tweets among topics.

**Figure 7:**
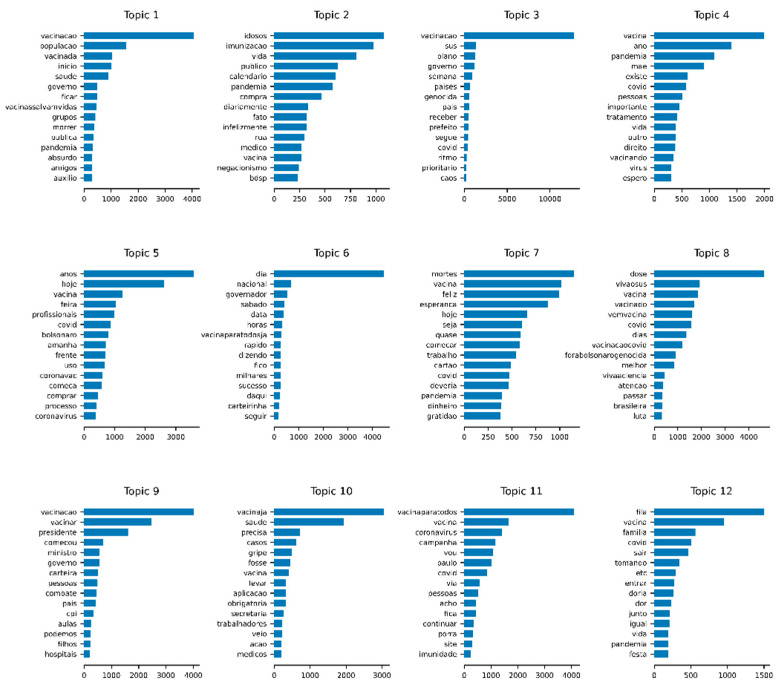

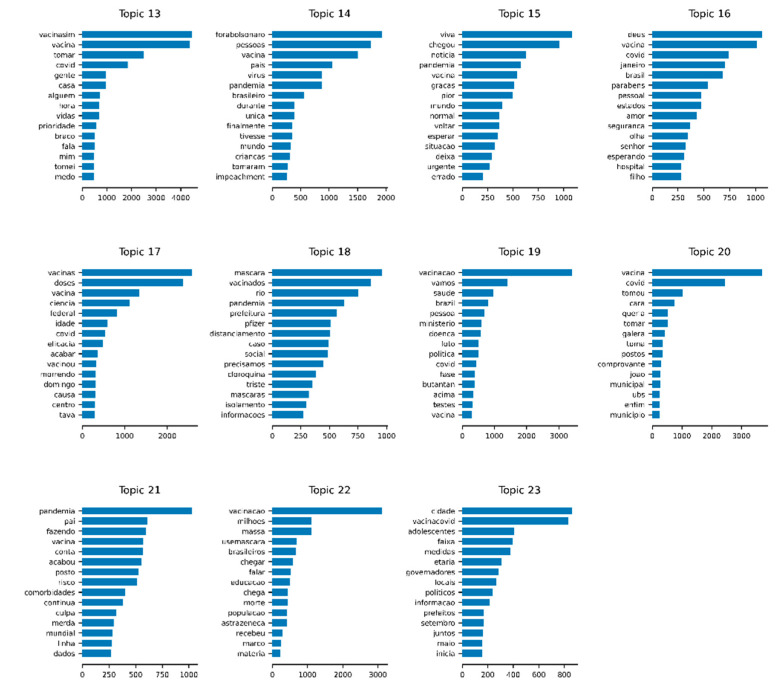
The fifteen terms with the highest weights in each topic.

For each topic, we also could extract the weights of the terms presented in Table S4 in the Supplementary Materials. Figure 7 presents the plot of the 15 terms with the highest weights in each topic (see the translations to English in Table S5).

**Figure 8:**
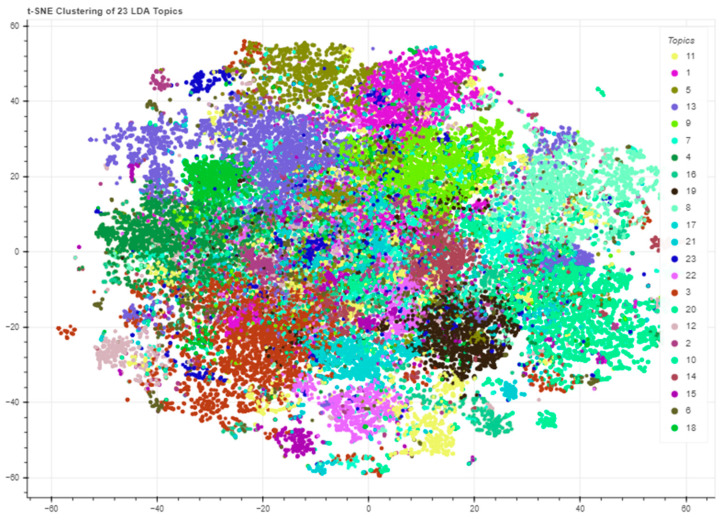
Tweets’ clusters in a two-dimensional plane, according to the topics they are related.

The segregation of clusters of tweets according to related topics was generated from the results of the *t*-SNE method, allowing another kind of visualization of the topics’ distributions in a two-dimensional projection. These clusters are shown in Figure 8.

#### 3.3.1. Manual Annotation Results

The manual annotation of topics according to the three defined polarities did not generate ties for most topics. Only two topics (9 and 17) presented label ties as each of the three annotators applied a different label. A fourth annotator (an Engineering researcher) was invited to support resolving the indecision in the two topics. The results of the polarity label annotation are shown in Table 1.

After resolving the ties, the labels’ distribution defined fifteen pro-vaccination topics, five neutral, and only three anti-vaccination. These labels enable the visualization of opinion distributions associated with the topics in space and time, helping to complete the analysis of the support and trust orientation regarding the COVID-19 immunization program in Brazil. After applying these labels to the corpus, the tweets’ counts were: 41,868 pro-vaccination, 9725 neutral, and 4165 anti-vaccination.

#### 3.3.2. Topics Timeline

The topic extraction process allowed the determination of the timelines for each of the 23 topics according to the number of tweets posted over the 17 months. As we obtained the topic → tweet association, it was possible to compute the totals of tweets collected according to each day for each topic.

**Figure 9:**
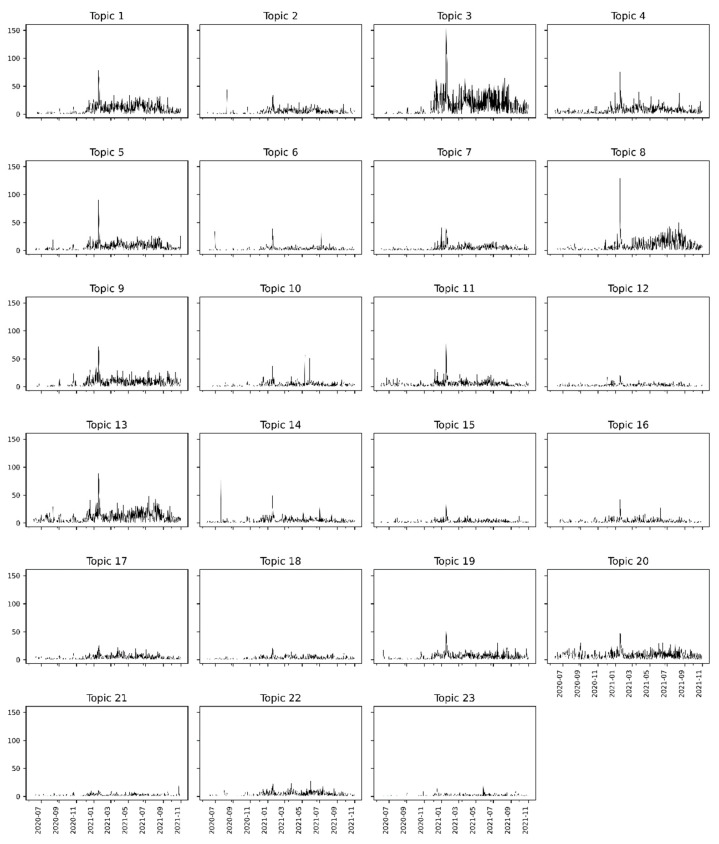
Timeline for each extracted topic, counting the number of tweets per day within the considered period.

The graph grid in Figure 9 presents the timelines, sharing the same axes (for the *x*-axis, the dates, and the *y*-axis, the tweets count). Each topic showed different peaks in the number of tweets on specific dates, with the highest peak in Topic 3 between January and February 2021, specifically on January 17, which is in line with the peak in the overall timeline of tweets (see Figure 2). Several smaller peaks in this same period can be observed in all other topics, whether they are the most significant peaks or not when we look inside each one.

The topic analysis over the seventeen months showed the trend of a significant peak in the count of tweets on several topics between January and February 2021. On 17 January 2021, the first vaccine application was carried out in Brazil, which had repercussions on social media and may be associated with this peak. Among the topics with peaks in this period, Topic 13 had the highest among all peaks, reaching 150 tweets.

All graphs show a trend toward fewer tweets between June and December 2020. As of December, the number of tweets on each topic started to grow. The month of December was marked by the launch of the National Vaccination Campaign against COVID-19, precisely on 16 December 2020. Therefore, from that date onwards, an intensification of online discussions on the subject was expected.

It is interesting to note that in the period between June and December 2020, some topics had salient peaks in the number of tweets: Topic 2 between August and September 2020, Topic 6 between June and July 2020, and Topic 14 between July and August 2020. These peaks may indicate, for instance, periods of interest in more specific investigations, supporting the discovery of public discussion trends.

**Figure 10:**
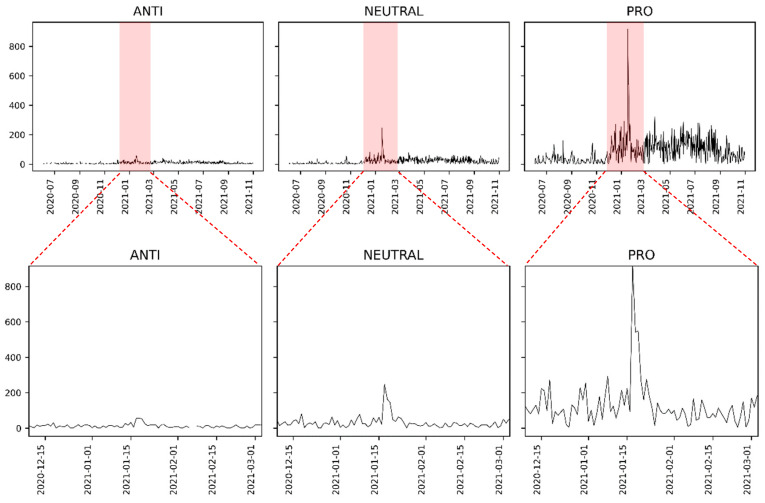
Timeline for each polarity, counting the number of tweets per day within the considered period.

It was possible to create a timeline for each label based on the association between polarities and topics. The graph grid in Figure 10 presents these timelines.

The graphs show that most tweets are pro-vaccination according to the topic-polarity association. Each polarity presented a most significant peak in the tweets number which we zoomed to show more detail. Note that the peaks occurred on 17 January 2021.

#### 3.3.3. Topics in Brazilian Geographic Space

The topic analysis in a geographic space has several levels of scope, ranging from the analysis of cities and neighborhoods to entire regions of a country. The granularity of the geographic information collected through the tweets allowed us to reach the municipal level, as seen in Figure 3.

Altogether, tweets from 3917 cities were retrieved, but not all of them had all the associated topics. It is also important to remember that only twenty-five cities had at least 300 tweets. As it is impossible to analyze the geographic distribution of all twenty-three topics in a shorter and more objective text such as this, we selected the two with the highest number of tweets to exemplify the analysis. Figures S1–S21 in the Supplementary Materials present the other topics’ spatial distributions.

**Figure 11:**
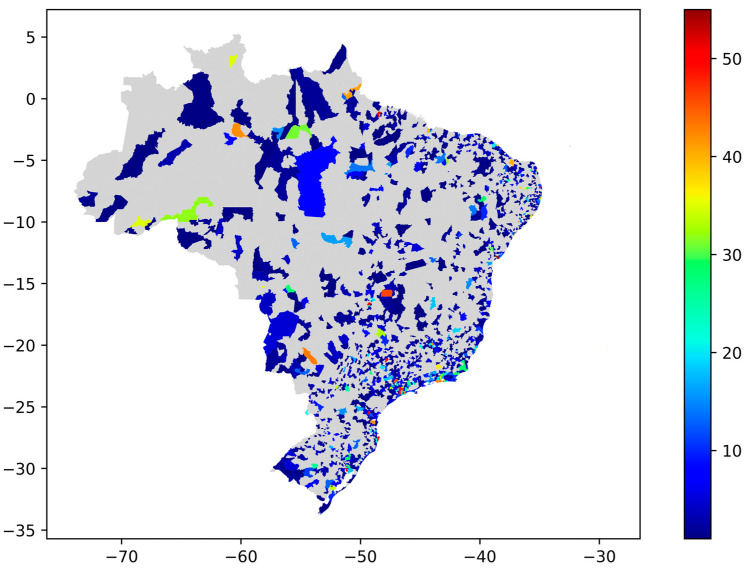
Topic 3 distribution on the Brazilian territory.

Topic 3, “Vaccination as a priority government action to combat COVID-19”, with pro-vaccination polarity, has the most significant number of tweets (n = 7165). The map in Figure 11 presents the distribution of Topic 3 in Brazilian cities. In the areas with darker red, there are the highest amounts of tweets from Topic 3.

**Figure 12:**
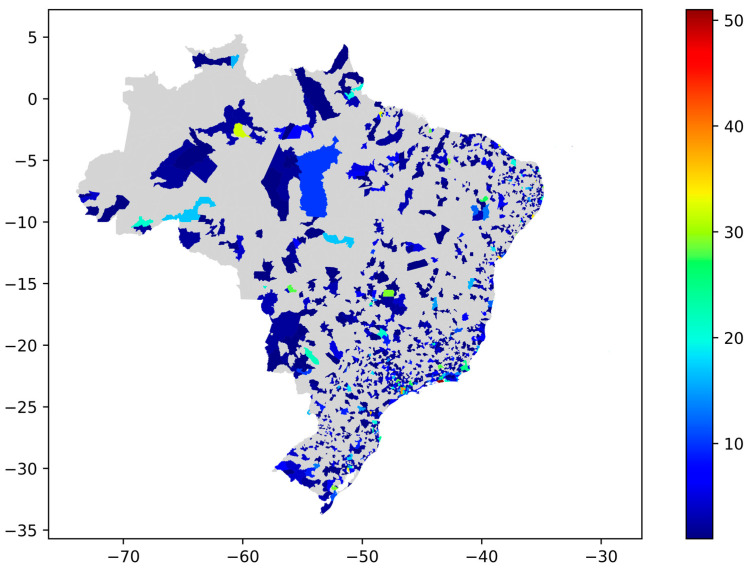
Topic 13 distribution on the Brazilian territory.

Topic 13, about “Vaccine saving lives concerning COVID-19” also with pro-vaccination polarity, has the second most significant number of tweets (n = 4815). Figure 12 contains the distribution of this topic in the Brazilian territory.

**Figure 13:**
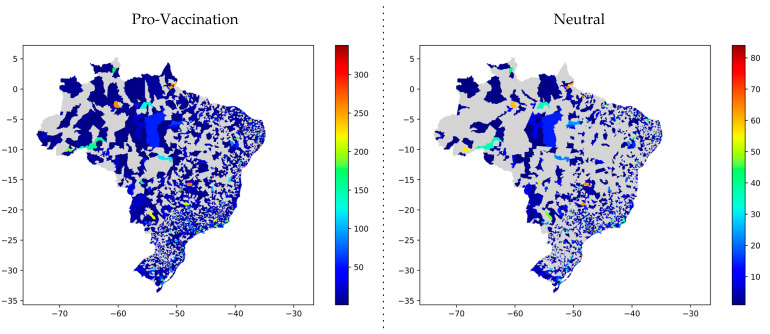

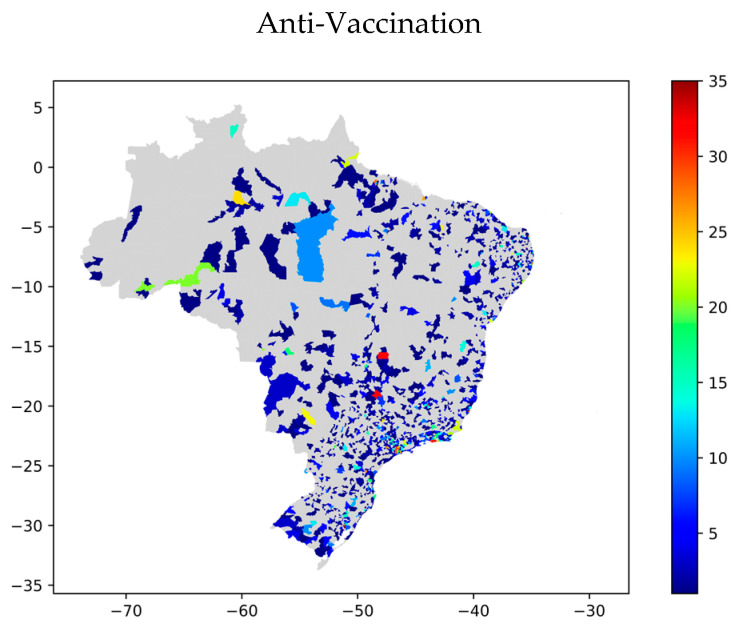
Distribution of tweets throughout the Brazilian territory according to polarities.

From the topic polarity annotation, in addition to analyzing the evolution of these polarities over 17 months, it is possible to visualize the geographic distribution of tweets according to the labels applied. The grid in Figure 13 contains maps according to polarity, making the distribution of tweets per city.

It was also possible to extract the ten cities with the highest number of tweets on each map according to polarities. Table 2 details these cities, separating them according to the polarity labels and presenting the tweets counting and percentual distributions. The populations presented for the cities came from the last population census of the Brazilian Institute of Geography and Statistics, which occurred in 2010.

The data in Table 2 highlights that São Paulo and Rio de Janeiro (both capitals of homonymous states and the two biggest cities in terms of population) had the highest number of pro-vaccination and neutral tweets. However, compared to other cities, which have smaller populations, they also have the smallest distribution of the amounts according to the population size, as seen through the percentual distribution column in the table. In the anti-vaccination polarity, Porto Alegre (capital of Rio Grande do Sul) had the highest number of tweets and the third highest percentual distribution score. São Paulo, followed by Rio de Janeiro, had the smallest distribution scores for the same polarity.

The percentual distribution scores represent interesting information to understand the opinions’ penetration according to each city’s population size. In cities with large populations, such as São Paulo and Rio de Janeiro, which also had some of the highest numbers of tweets, it is noted lower percentual distributions of tweets, a fact that may indicate that the extension of opinion effects is smaller than in cities with smaller populations and large amounts of tweets.

For example, looking at the pro-vaccination polarity, the city with the smallest population, São Luís (capital of the state of Maranhão), had a higher percentual distribution than São Paulo and Rio de Janeiro, indicating that the opinions may have had better penetration. Regarding opinions supporting vaccination, it is potentially desirable for the agencies managing the programs.

On the other hand, Porto Alegre, the city with the second highest population in the southern Brazilian region and the highest number of anti-vaccination tweets, had the third highest percentual distribution, demonstrating a greater tendency for penetration of negationist opinions. Still within the anti-vaccination polarity, the city of Vila Velha, despite having the smallest population among the ten cities listed, has the highest percentual distribution score, indicating that there may be a potential domain of negationist opinions shared by Twitter users.

Considering the ten cities with the highest number of tweets for each polarity, we can see that all cities are state capitals for the pro-vaccination polarity. In the neutral polarity, there is a predominance of capitals, except for two cities: Campinas and Uberlândia. Campinas has the third-largest population in the state of São Paulo, and Uberlândia has the second-largest population in the state of Minas Gerais. Finally, for the anti-vaccination polarity, there is also a predominance of the capital, except for two cities: Uberlândia and Vila Velha. Vila Velha is the second largest city in terms of population of the state of Espirito Santo, even ahead of the state capital, Vitória, which does not appear in Table 2.

### 3.4. Theoretical and Practical Implications

From a theoretical point of view, our study provides a methodological approach to analyze topics in space and time, using data obtained from Twitter about the Brazilian COVID-19 immunization program. However, the methodology was designed to be independent of research themes, contributing to the development of other research to understand how public opinion on the subject of interest evolves in space and time. There are other methods specifically for topic modeling and extraction; however, we rely on the literature when using LDA (see, for instance, [10,15,57,58,59]) since it corrects problems that occur in other similar methods, enabling us to achieve our study’s objectives.

From a practical point of view, this work follows the same line as de Carvalho et al. [33]. It intends to present solutions for agencies and authorities related to immunization campaigns, to carry out analyzes to identify foci of misinformation, applying measures to mitigate the damage caused in the long-term public opinion. We presented a toolbox with a coherent and well-interconnected stepwise to generate reliable results in combating misinformation. This toolbox is a highlighted practical implication in infodemic-related research [60].

Authorities focused on combating this negative aspect of the large data volumes have the potential benefits of understanding the opinions distribution, the topics discussed, and analyzing the evolution of these discussions from the desired point in time, in addition to identifying which locations generate the most misinformation. Identifying which entities (people or organizations) are responsible for creating and spreading misinformation also becomes possible, delimiting the analysis to more particular levels. Based on these last comments, the infodemic opening space for cybercrimes or generating situations of civil danger is an issue to be explored by further studies [61,62].

In this context, while in our previous study we dealt only with the opinion classification based on a textual training dataset containing tweets with pro, anti-vaccination, or neutral orientation, manually searching for correlated events on social web and news search engines, in the current work we sought to deepen the investigation with new elements. We identified the main topics discussed so that researchers from different areas can apply manual annotation of polarity labels, no longer using machine classification, but now classification based on human judgment, for later automation of the hierarchical association *polarity → topic → tweet*.

From the previous work, we only used two sets of informational elements that we consider fundamental: (1) a part of one of the textual bases that contained geocoded tweets so that we could expand our developments with the distribution of topics in the Brazilian geographic space; and (2) the three-category classification scheme, with the pro, anti, and neutral labels.

The framework in the current study used different models than the previous one, although they were machine learning models in both cases. The present study applied topic modeling, using the Latent Dirichlet Allocation, and consequently clustering for visualization, with the *t*-SNE, enabling a more detailed view of the distribution of topics and polarities, according to the number of tweets, in time and Brazilian geographic space. In contrast, our previous work applied only classification models allowing the polarities evolution over time. The use of topic modeling was fundamental to increasing the specificity of the results obtained concerning the previous study.

One last implication to be mentioned is the development of new epidemiologic analyses of the COVID-19 outbreak [63]: infodemic and misinformation analysis can bring exciting findings about how people’s opinions evolved in association with the evolution of the disease, using the numbers of infections and deaths over the time, for instance, as parameters to be associated with the collected opinion and related trust. It also should be noted that although we have dedicated the study to vaccination against COVID-19 in Brazil, the analytical framework can be used for other purposes in other domains, considering the guarantee of public health and security. We understand that even the private sector can use this process, for example, to understand how opinions about brands or products evolve over time and in a given geographic space.

### 3.5. Difficulties, Challenges, and Limitations

Although our methodological approach is based on techniques and tools implemented through secure and robust software, there is a need for subsequent refinements, especially in the text preprocessing and the definition and extraction of the features used in the process. There are several difficulties involved, listing some of them: we need to deal with texts outside the formal standard of the Portuguese language, which generates several noises such as abbreviations, the use of emojis/emoticons, and even the expansion of the stop words lists to include also associated abbreviations, and grammatical errors [64].

In this way, the construction of preprocessing scripts becomes challenging [65], even though there are already methods implemented in libraries and frameworks such as those applied in this study. There is a need to define a viable strategy for texts to be processed and transformed into a workable format by topic modeling methods. There are important decisions to be made about what should remain in the text and what should be removed, especially if we consider the indication made by Silva et al. [66] to keep the text as close as possible to the usage pattern from which it was extracted.

Another difficulty is related to the geocoding of tweets. We extracted geographic information as described in the methodological section, but we need to complete the information about the coordinates through points with latitude and longitude using Google’s geographic API. The geographic information we could extract directly via the Twitter API brought us polygonal coordinates when our objective was the points referring to cities. As we also obtained the names of cities through Twitter API, we developed a process that retrieves the geographic points referring to these cities via Google API. Furthermore, the number of tweets obtained with geocoding was much lower than the number of tweets we parameterized to be searched and retrieved, as we reported in our previous work [33], implying that we obtained all the geocoded tweets according to the defined scraping parameters.

Thus, a critical limitation was the low number of geocoded tweets, with information from specific points to determine which cities generated the posts. It is crucial to retrieve more extensive amounts of geocoded tweets, to ensure statistical significance to the corpus to train topic extraction methods and consequently to ensure reliable analyses and results. Using tweets containing geographic information allows new studies to apply methods for analyzing correlations between variables identified through the texts with the variables traditionally used by management agencies, as in the case of vaccination programs. For example, the influence that pro- and anti-vaccination texts can exert on the flow of people receiving vaccinations can be studied using methods such as those applied by Huang et al. [67].

Even though the LDA fixes existing problems in methods prior to it [68], there are criticisms of its performance when applied to short texts, caused by the sparseness problem, which occurs with this kind of text [69]. Other models, more suitable for short texts, can be used as substitutes for the LDA in our framework: the Dual-sparse Topic Model (Dsparse TM) proposed by Lin et al. [70], the Biterm Topic Model (BTM) proposed by Cheng et al. [71], and the Pseudo-document-based Maximum entropy discrimination Latent Dirichlet Allocation model (PSLDA) proposed by Sun et al. [72] are three state-of-art examples.

Another issue that still needs further development concerns the psychological effects of COVID-19 and related issues on people and the way they manifest on the social web. In this study, although we are considering people’s opinions, we have not developed any analysis of psychological effects in parallel with the study of opinions. Several studies have demonstrated the need to investigate the effects of the pandemic on people’s mental health, evaluating, for example, effects such as anxiety and the causes of hesitation to receive the vaccine (see, for instance, [23,31,73,74]).

Although our study sought the judgments of researchers from different areas to act as polarity labels annotators, there are still many gaps about this type of annotation to be addressed, including the annotators’ suggestions for refining the process we intend to follow for future research. Using only three labels on topic polarity also restricted the scope of related analyses. A more extensive list of labels referring to the emotions perceived by the annotators concerning the topics can improve the analyses.

## 4. Final Considerations

This article presented a study developed to identify topics discussed on Twitter between 2020 and 2021 about the COVID-19 immunization program in Brazil. The main contribution of the research was the definition of a framework containing a set of tools to deliver structured and helpful information, supporting agencies and authorities involved in the immunization program to understand the evolution of public opinion on vaccination and identifying cities with significant numbers of posts according to the extracted topics.

The methodology considered a manual annotation process on the topics, attributing polarity labels concerning support and trust in vaccination. Based on this labeling, it was possible to present both the evolution of polarities over time and their geographic distribution in Brazilian cities.

Interesting results were obtained regarding information surveillance (or infoveillance) about vaccination on social networks in the infodemic context, which can help guide efforts to combat potentially harmful information (or misinformation, as we have been calling it) to society and vaccination programs.

The temporal evolution analysis allowed the visualization of the moment when Brazilian posts started intensifying on the explored theme: from the launch of the national vaccination campaign in December 2020. It was also possible to detect when the highest peak of posts occurred: on 17 January 2021, when the first vaccine was applied in Brazil. There was a tendency for most of the topics to present peak on the same date.

Regarding the evolution of tweets for polarities, the peak on 17 December 2021, was more marked in the pro-vaccination orientation. However, they are also perceived in the anti-vaccination and neutral polarities on minor scales.

In the analysis of the distribution in the Brazilian geographic space, a greater concentration of tweets was identified in large cities, especially in the two largest cities in the country: São Paulo and Rio de Janeiro. However, based on the data collected, these two cities also had the lowest tweet percentual distributions according to their populations. The spatial analysis of the hierarchical association polarity → topic → tweet allowed the observation of the potential of some cities concerning the penetration of each opinion polarity in their populations.

It is worth mentioning that any other immunization program for other diseases can use the methodological approach presented. If we further expand this vision, the agencies involved in managing any crisis in any critical public sector are potential methodology users.

### Further Research

As directions for further research, we highlight: (1) improvement of the technical data scraping process to extract more significant amounts of geocoded tweets about the desired topic; (2) development of an improved process for manual annotation on the opinion polarities, also considering labels referring to emotions such as happiness, anger, anguish, anxiety, fear, sadness, surprise, etc. Additionally, we highlight [75] for new space and time analyses; (3) testing and comparing the performance of other topic modeling algorithms specifically designed to work with short texts; (4) combining prospects of social distancing and mobility restrictions in different categories within the geographical locations [76]; and (5) analysis from the perspective of the psychological effects of public discussion on the social web on how the individual develops his opinion on the immunization programs.

## Figures and Tables

**Table 1:** Results of the manual annotation process.

Topic	Label	Topic	Label
1	Pro	13	Pro
2	Pro	14	Pro
3	Pro	15	Neutral
4	Pro	16	Neutral
5	Neutral	17	Anti
6	Pro	18	Anti
7	Pro	19	Pro
8	Pro	20	Pro
9	Neutral	21	Neutral
10	Pro	22	Pro
11	Pro	23	Pro
12	Anti	-	-

**Table 2:** Tweets’ counts and percentual distributions for each of the ten cities with the highest number of tweets by polarity.

Pro				Neutral			
City	Population	Count	Distribution * (%)	City	Population	Count	Distribution * (%)
São Paulo	11,253,503	338	0.00300	São Paulo	11,253,503	84	0.00075
Rio de Janeiro	6,320,446	321	0.00508	Rio de Janeiro	6,320,446	78	0.00123
Curitiba	1,751,907	279	0.01593	Belém	1,393,399	67	0.00481
Salvador	2,675,656	276	0.01032	Campinas	1,080,113	66	0.00611
São Luís	1,014,837	273	0.02690	Fortaleza	2,452,185	65	0.00265
Belo Horizonte	2,375,151	269	0.01133	Florianópolis	421,240	64	0.01519
Porto Alegre	1,409,351	267	0.01894	Brasília	2,570,160	63	0.00245
Goiânia	1,302,001	266	0.02043	Uberlândia	604,013	63	0.01043
Brasília	2,570,160	265	0.01031	Macapá	398,204	63	0.01582
Recife	1,537,704	265	0.01723	Salvador	2,675,656	62	0.00232
		**Anti**					
		**City**	**Population**	**Count**	Distribution * (%)		
		Porto Alegre	1,409,351	35	0.0025		
		Brasília	2,570,160	32	0.0012		
		Uberlândia	604,013	32	0.0053		
		Rio de Janeiro	6,320,446	31	0.0005		
		Belo Horizonte	2,375,151	30	0.0013		
		Aracaju	571,149	30	0.0053		
		Vila Velha	414,586	29	0.0070		
		São Paulo	11,253,503	29	0.0003		
		Recife	1,537,704	29	0.0019		
		Belém	1,393,399	28	0.0020		

* Defined in the form of percentages according to the population of each city.

## Data Availability

The corpora files are not publicly available because of Twitter’s Developer Agreement; however, we can make them available upon request, for academic research purposes.

## Supplementary Materials

The following supporting information can be downloaded at: https://www.mdpi.com/article/10.3390/tropicalmed7120425/s1, Table S1: Parameters used in the scraping script; Table S2: Example of the georeferenced corpus, containing the first five rows; Table S3: Cities with at least 300 tweets retrieved; Table S4: The 23 topics extracted using LDA algorithm; Table S5: The 23 topics terms translations (or approximated translations) to English; Table S6: Possible interpretations for each topic; Table S7: Tweets amounts according to each topic; Figure S1: Topic 1 distribution on the Brazilian territory; Figure S2: Topic 2 distribution on the Brazilian territory; Figure S3: Topic 4 distribution on the Brazilian territory; Figure S4: Topic 5 distribution on the Brazilian territory; Figure S5: Topic 6 distribution on the Brazilian territory; Figure S6: Topic 7 distribution on the Brazilian territory; Figure S7: Topic 8 distribution on the Brazilian territory; Figure S9: Topic 10 distribution on the Brazilian territory; Figure S10: Topic 11 distribution on the Brazilian territory; Figure S11: Topic 12 distribution on the Brazilian territory; Figure S12: Topic 14 distribution on the Brazilian territory; Figure S13: Topic 15 distribution on the Brazilian territory; Figure S14: Topic 16 distribution on the Brazilian territory; Figure S15: Topic 17 distribution on the Brazilian territory; Figure S16: Topic 18 distribution on the Brazilian territory; Figure S17: Topic 19 distribution on the Brazilian territory; Figure S18: Topic 20 distribution on the Brazilian territory; Figure S19: Topic 21 distribution on the Brazilian territory; Figure S20: Topic 22 distribution on the Brazilian territory; Figure S21: Topic 23 distribution on the Brazilian territory.
