# Supplementary material for: The COVID-19 Infodemic on Twitter: A Space and Time Topic Analysis of the Brazilian Immunization Program and Public Trust
Source: Trop Med Infect Dis. 2022 Dec 9;7(12):425. doi: 10.3390/tropicalmed7120425 (PMC9783210; doi:10.3390/tropicalmed7120425)
Supplement: Supplementary file 1 [file tropicalmed-07-00425-s001.zip › tropicalmed-2036132-supplementary.pdf]

Supplementary Materials

Tables

Table S1. Parameters used in the scraping script.

**Type of parameter**	**Details**
Keywords	1st block, COVID/pandemic related terms with hashtags: *#covid, #covid-19, #covid19, #covid_19, #coronavírus, #pandemia covid*.<br><br>2nd block, COVID/pandemic related terms without hashtags: *covid, covid-19, covid19, covid_19, coronavirus, pandemia*.<br><br>3rd block, COVID vaccination related terms with hashtags: *#vacina, #vacinacao, #vacinacovid-19, #vacina_corona, #vacina_covid, #vacina_covid-19, #vacina_covid_19, #vacina_coronavirus, #vacina_covid19, #vacinacaocorona, #vacinacaocovid-19, #vacinacaocovid_19, #vacinacaocoronavirus, #vacinacao_corona, #vacinacaocovid19, #vacinacao_covid, #vacinacao_coronavirus, #vacinacao_covid19, #vacinacao_covid-19, #vacinacao_covid_19*.<br><br>4th block, COVID vaccination related free terms: *vacina, vacinacao, vacinar, vacinado*.
Geographical constraint	*place_country:BR*, to retrieve only tweets from Brazil
Language constraint	*lang:pt*, to retrieve only tweets in Portuguese

Table S2. Example of the georeferenced corpus, containing the first five rows.

	text[1]	place_name	country	latitude[2]	longitude[2]	created_at
0	Vacina cubana 'Soberana', contra covid-19, ter...	Rio de Janeiro	Brasil	-22.906847	-43.172897	2020-12-31 23:47:35+00:00
1	#CORONAVÍRUS: Em reunião com TCE-AM, @Amazonas...	Manaus	Brasil	-3.119028	-60.021731	2020-12-31 23:47:08+00:00
2	Vou virar o ano isolado até da própria família...	Natal	Brasil	-5.784169	-35.199971	2020-12-31 23:16:48+00:00
3	@jose_simao Mas o problema não é o covid...e s...	São Paulo	Brasil	-23.555771	-46.639557	2020-12-31 23:10:50+00:00
4	Tomara que chova no Brasil inteiro pra não ter...	Santa Isabel do Ivaí	Brasil	-23.001704	-53.196903	2020-12-31 22:52:18+00:00

[1] The texts were left truncated in the table for spacing reasons, however, the texts used in the process were the complete versions.
[2] The geographic coordinate point was subdivided into two columns, one for latitude and one for longitude.

Table S3. Cities with at least 300 tweets retrieved.

City*	Count	City*	Count
São Paulo	451	Goiânia	338
Rio de Janeiro	430	Macapá	332
Salvador	361	Campinas	331
Brasília	360	Florianópolis	330
Belo Horizonte	356	João Pessoa	323
Belém	352	Natal	323
Curitiba	351	Teresina	319
Recife	345	Aracaju	318
Niterói	345	Maceió	310
Porto Alegre	344	Santos	303
São Luís	343	Vitória	303
Fortaleza	342	Uberlândia	300
Manaus	341		

* Cities highlighted in grey are Brazilian states' capitals.

Table S4. The 23 topics extracted using LDA algorithm.

	Term 1	Term 2	Term 3	Term 4	Term 5	Term 6	Term 7	Term 8	Term 9	Term 10	Term 11	Term 12	Term 13	Term 14	Term 15
**Topic 1**	vacinacao	populacao	vacinada	inicio	saude	governo	ficar	vacinassalvamvidas	grupos	morrer	publica	pandemia	absurdo	amigos	auxilio
**Topic 2**	idosos	imunizacao	vida	publico	calendario	pandemia	compra	diariamente	fato	infelizmente	rua	medico	vacina	negacionismo	bdsp
**Topic 3**	vacinacao	sus	plano	governo	semana	paises	genocida	pais	receber	prefeito	segue	covid	ritmo	prioritario	caos
**Topic 4**	vacina	ano	pandemia	mae	existe	covid	pessoas	importante	tratamento	vida	outro	direito	vacinando	virus	espero
**Topic 5**	anos	hoje	vacina	feira	profissionais	covid	bolsonaro	amanha	frente	uso	coronavac	comeca	comprar	processo	coronavirus
**Topic 6**	dia	nacional	governador	sabado	data	horas	vacinaparatodosja	rapido	dizendo	fico	milhares	sucesso	daqui	carteirinha	seguir
**Topic 7**	mortes	vacina	feliz	esperanca	hoje	seja	quase	comecar	trabalho	cartao	covid	deveria	pandemia	dinheiro	gratidao
**Topic 8**	dose	vivaosus	vacina	vacinado	vemvacina	covid	dias	vacinacaocovid	forabolsonarogenocida	melhor	vivaaciencia	atencao	passar	brasileira	luta
**Topic 9**	vacinacao	vacinar	presidente	comecou	ministro	governo	carteira	pessoas	combate	pais	cpi	aulas	podemos	filhos	hospitais
**Topic 10**	vacinaja	saude	precisa	casos	gripe	fosse	vacina	levar	aplicacao	obrigatoria	secretaria	trabalhadores	veio	acao	medicos
**Topic 11**	vacinaparatodos	vacina	coronavirus	campanha	vou	paulo	covid	via	pessoas	acho	fica	continuar	porra	site	imunidade
**Topic 12**	fila	vacina	familia	covid	sair	tomando	etc	entrar	doria	dor	junto	igual	vida	pandemia	festa
**Topic 13**	vacinasim	vacina	tomar	covid	gente	casa	alguem	hora	vidas	prioridade	braco	fala	mim	tomei	medo
**Topic 14**	forabolsonaro	pessoas	vacina	pais	virus	pandemia	brasileiro	durante	unica	finalmente	tivesse	mundo	criancas	tomaram	impeachment
**Topic 15**	viva	chegou	noticia	pandemia	vacina	gracas	pior	mundo	normal	voltar	esperar	situacao	deixa	urgente	errado
**Topic 16**	deus	vacina	covid	janeiro	brasil	parabens	pessoal	estados	amor	seguranca	olha	senhor	esperando	hospital	filho
**Topic 17**	vacinas	doses	vacina	ciencia	federal	idade	covid	eficacia	acabar	vacinou	morrendo	domingo	causa	centro	tava
**Topic 18**	mascara	vacinados	rio	pandemia	prefeitura	pfizer	distanciamento	caso	social	precisamos	cloroquina	triste	mascaras	isolamento	informacoes
**Topic 19**	vacinacao	vamos	saude	brazil	pessoa	ministerio	doenca	foto	politica	covid	fase	butantan	acima	testes	vacina
**Topic 20**	vacina	covid	tomou	cara	queria	tomar	galera	toma	postos	comprovante	joao	municipal	ubs	enfim	municipio
**Topic 21**	pandemia	pai	fazendo	vacina	conta	acabou	posto	risco	comorbidades	continua	culpa	merda	mundial	linha	dados
**Topic 22**	vacinacao	milhoes	massa	usemascara	brasileiros	chegar	falar	educacao	chega	morte	populacao	astrazeneca	recebeu	marco	materia
**Topic 23**	cidade	vacinacovid	adolescentes	faixa	medidas	etaria	governadores	locais	politicos	informacao	prefeitos	setembro	juntos	maio	inicia

Table S5. The 23 topics terms translations (or approximated translations) to English.

	Term 1	Term 2	Term 3	Term 4	Term 5	Term 6	Term 7	Term 8	Term 9	Term 10	Term 11	Term 12	Term 13	Term 14	Term 15
**Topic 1**	vaccination	people	vaccinated	start	health	government	to stay	vaccinessavelives	groups	to die	public	pandemic	absurd	friends	aid
**Topic 2**	elderly people	immunization	life	public	calendar	pandemic	purchase	daily	fact	unfortunately	street	doctor	vaccine	negationism	bdsp (Portuguese acronym for "good morning São Paulo")
**Topic 3**	vaccination	sus (Portuguese acronym for "Brazilian Public Health System”)	plan	government	week	countries	genocidal	country	to receive	mayor	follow	covid	rhythm	priority	chaos
**Topic 4**	vaccine	year	pandemic	mother	exists	covid	people	important	treatment	life	other	right	vaccinating	virus	[I] expect
**Topic 5**	years	today	vaccine	fair/market	professionals	covid	Bolsonaro (a family name related to the Brazilian president)	tomorrow	front	usage	coronavac	begins	purchase	process	coronavirus
**Topic 6**	day	national	governor	saturday	date	hours	vaccineforeverybodynow	fast	saying	[I] stay	thousands	success	from here	little card	follow
**Topic 7**	deaths	vaccine	happy	hope	today	be	almost	to start	job	card	covid	should	pandemic	money	gratitude
**Topic 8**	shot	hailthesus (something like “Hail the Brazilian Public Health System”)	vaccine	vaccinated	comevaccine	covid	days	covidvaccination	outgenocidalbolsonaro	best	longlivescience	warning	to pass	brazilian	fight
**Topic 9**	vaccination	to vaccinate	president	started	minister	government	card	people	combat	country	cpi (Portuguese acronym for “Parliamentary Committee of Inquiry”)	classes	[we] can	children	hospitals
**Topic 10**	vaccinenow	health	need	cases	the flu	were	vaccine	to take	application	mandatory	secretariat	workers	came	action	doctors
**Topic 11**	vaccineforeverybody	vaccine	coronavirus	campaign	I will/I am going	paulo (the proper name “Paul”, in Portuguese)	covid	via (it can be translated as “way”, or as a conjugation of the verb “to see” like “saw”)	people	[I] think	stay	to continue	porra (it is a popular low-slang interjection in Portuguese)	site	immunity
**Topic 12**	queue	vaccine	family	covid	to leave	taking	etc	enter	doria (João Doria, a Brazilian	pain	together	equal	life	pandemic	party

									businessman and politician)						
**Topic 13**	vaccineyes	vaccine	to take	covid	people	house	somebody/someone/anybody	hour	lives	priority	arm	speech	me/myself	[I] took	fear
**Topic 14**	outbolsonaro	people	vaccine	country	virus	pandemic	brazilian	during	only	finally	had	world	children	[they] took	impeachment
**Topic 15**	hail	has arrived	news	pandemic	vaccine	thanks	worst	world	regular	to come back	to wait	situation	leaves	urgent	wrong
**Topic 16**	god	vaccine	covid	january	brazil	congratulations	personnel	states	love	security	look	mister	waiting	hospital	son
**Topic 17**	vaccines	shots	vaccine	science	federal	age	covid	efficiency	to finish	vaccinated	dying	sunday	cause	center	was
**Topic 18**	mask	vaccinated	rio (it can mean “river” in Portuguese, or can refer to the city of “Rio de Janeiro”)	pandemic	town hall	pfizer	distancing	case	social	[we] need	chloroquine	sad	masks	isolation	information
**Topic 19**	vaccination	[we] will	health	brazil	person	ministry	disease	picture	policy	covid	stage/phase	butantan (referring to the Butantan Institute, which produces vaccines in Brazil)	above	tests	vaccine
**Topic 20**	vaccine	covid	took	cara (in colloquial usage it can mean “guy”, but it can also mean “face”)	[I] wanted	to take	crowd	take	[health] centers (referring to the “health centers” where the vaccines are applied)	certificate	joao (the proper name “John” in Portuguese)	municipal	ubs (Portuguese acronym for “basic health unit”)	anyway	municipality
**Topic 21**	pandemic	father	making/doing	vaccine	account	ended	[health] centers (referring to the “health centers” where the vaccines are applied)	risk	comorbidities	continue	fault	merda (it is a popular low-slang interjection in Portuguese)	worldwide	line	data
**Topic 22**	vaccination	millions	mass	usemask	brazilian	to arrive	to speak	education	It can mean “enough!” (an interjection) or “[he] arrives”	death	population	astrazeneca	received	milestone	matter/substance
**Topic 23**	city	covidvaccine	teenagers/adolescents	range	measures	age	governors	places	politicians	information	mayors	september	together	may	starts

Table S6. Possible interpretations for each topic.

Topic	Interpretation	Topic	Interpretation
1	Start of vaccination for the population by the government to contain the pandemic	13	Vaccine saving lives concerning COVID-19
2	Immunization for elderly people	14	Vaccination against COVID-19 and potential impeachment of the president of Brazil
3	Vaccination as a priority government action to combat COVID-19	15	Arrival of vaccines to bring normality concerning the terrible situation generated by the pandemic
4	Vaccination as a right of the people to fight COVID-19	16	Brazilian states vaccinating against COVID-19 from January*
5	Beginning of the process of purchasing vaccines against COVID-19 by the Brazilian government	17	Questioning the effectiveness of vaccines against COVID-19, associating them with the deaths of people who took vaccines shots
6	National vaccination campaign serving thousands of people	18	Criticism of isolation measures and vaccination with mention of the use of medication without proven efficacy against the disease
7	Vaccines bringing hope for reducing the number of deaths in the pandemic	19	Call for vaccination against COVID-19, with tested and approved vaccines in Brazil
8	Support to the Brazilian Public Health Service (SUS) and vaccination	20	People go to health centers to be vaccinated and receive the cards that prove the vaccination
9	Vaccination process started, with the action of politicians, seeking to fight the disease to promote the return to school and the relief of hospitals	21	Vaccines are being produced worldwide to combat the pandemic and considering the risks associated with comorbidities
10	Mandatory vaccination for workers considering cases of flu	22	Support for vaccination against COVID-19, the use of masks, and the education of Brazilians to prevent deaths
11	COVID-19 vaccination campaign to ensure immunity for all people	23	Start of vaccination of the adolescent public age groups
12	People facing queues to get the COVID-19 vaccine		

*The first COVID-19 vaccine was applied in Brazil on January 17, 2021

Table S7. Tweets amounts according to each topic.

Topic	Tweets Amount	Topic	Tweets Amount
1	3,970	13	4,815
2	1,867	14	1,836
3	7,165	15	1,127
4	3,295	16	1,359
5	3,095	17	1,803
6	1,075	18	1,297
7	1,872	19	2,387
8	3,804	20	3,415
9	3,196	21	948
10	1,515	22	1,728
11	2,380	23	744
12	1,065	Total	55,758

Figures

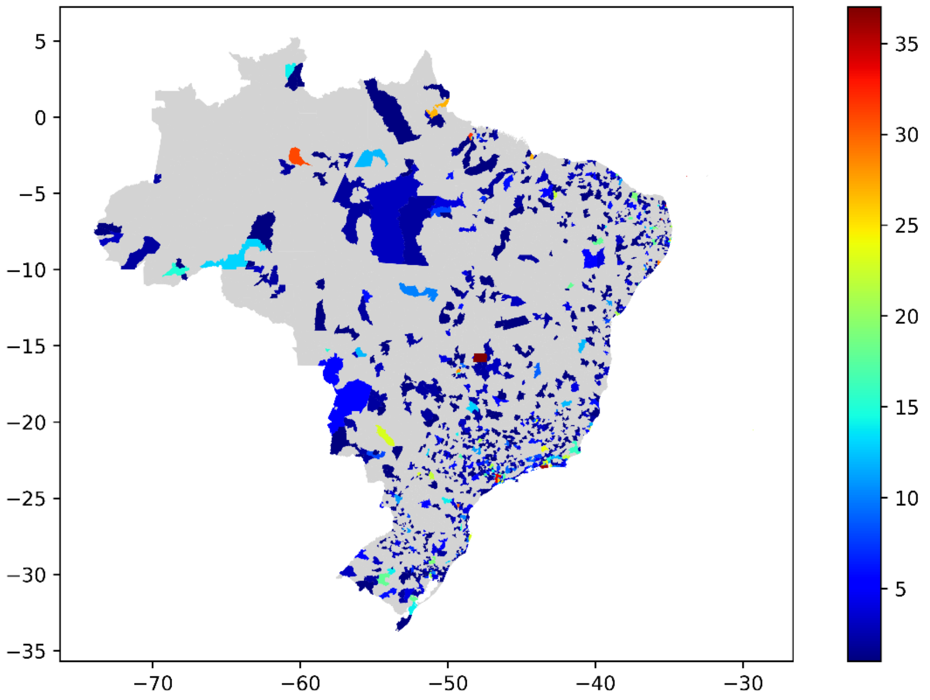

Figure S1. Topic 1 distribution on the Brazilian territory.

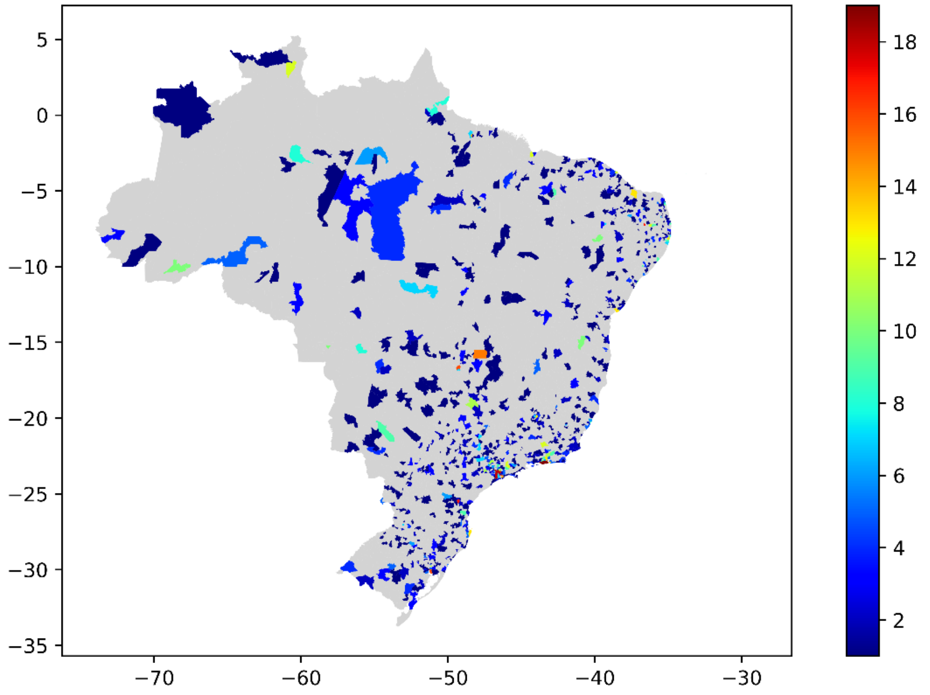

Figure S2. Topic 2 distribution on the Brazilian territory.

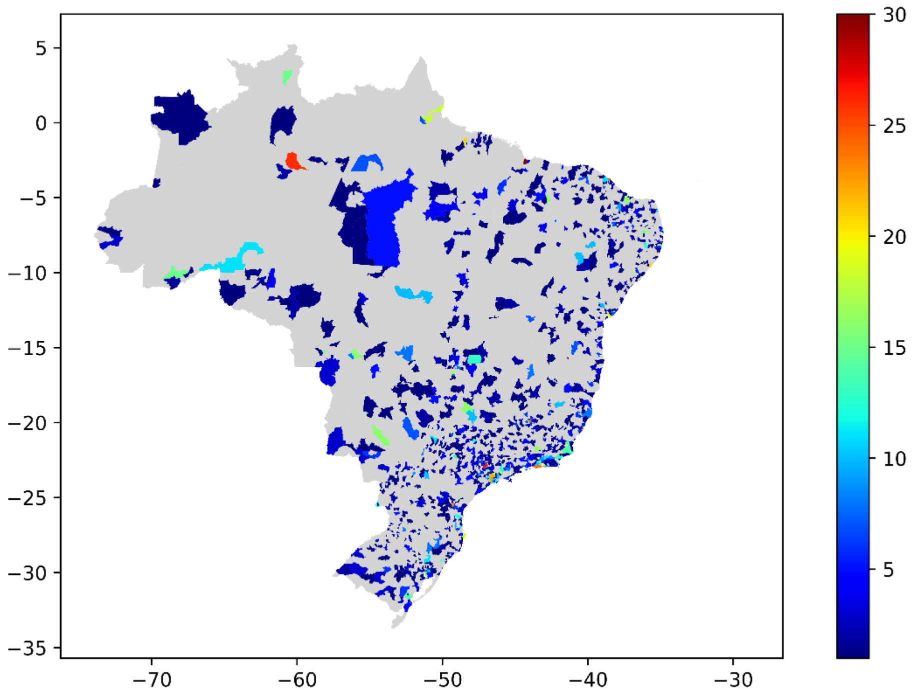

Figure S3. Topic 4 distribution on the Brazilian territory.

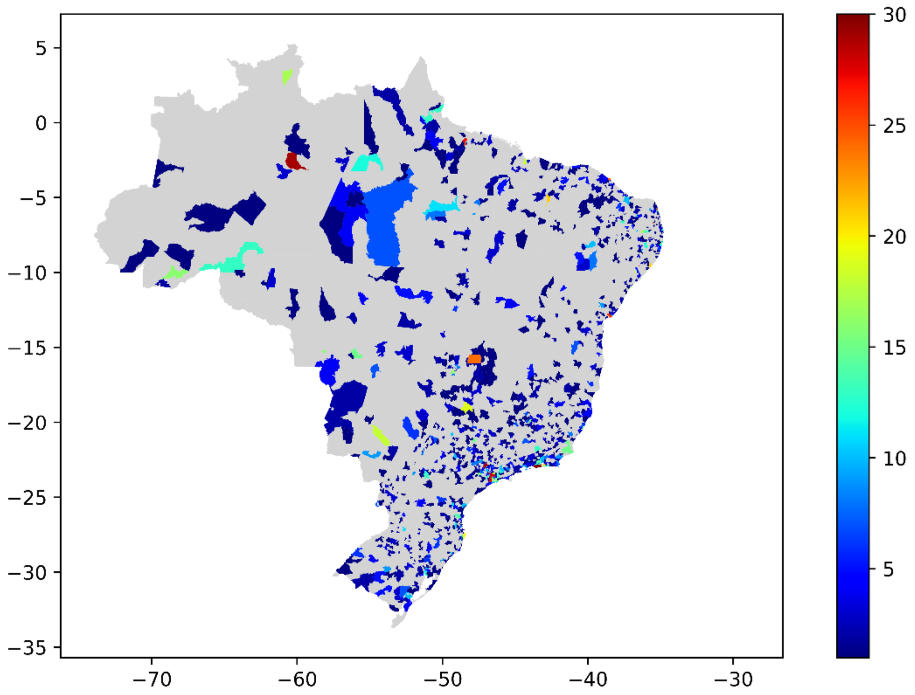

Figure S4. Topic 5 distribution on the Brazilian territory.

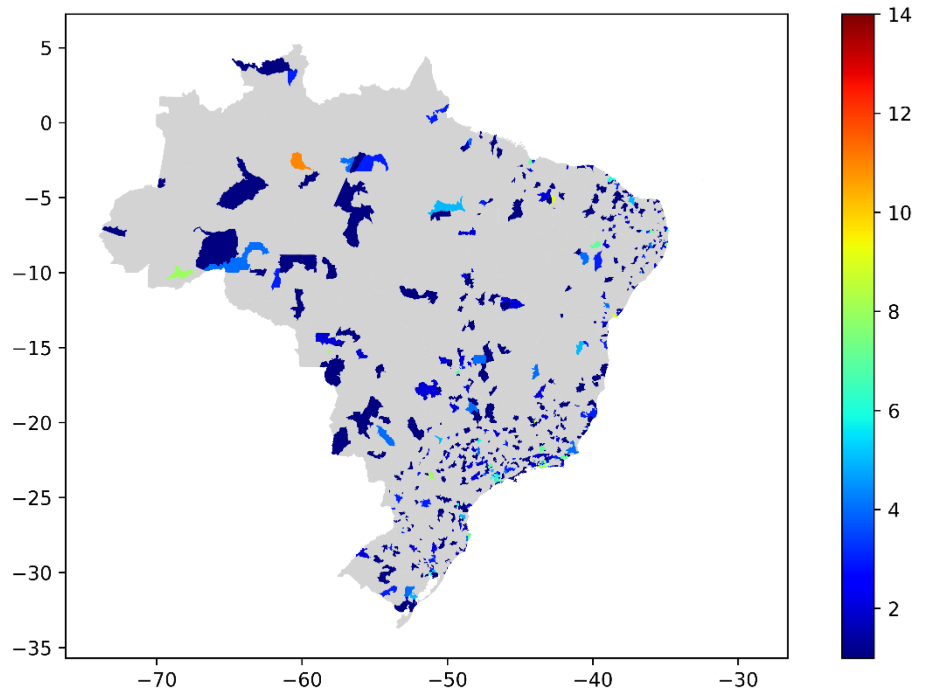

Figure S5. Topic 6 distribution on the Brazilian territory.

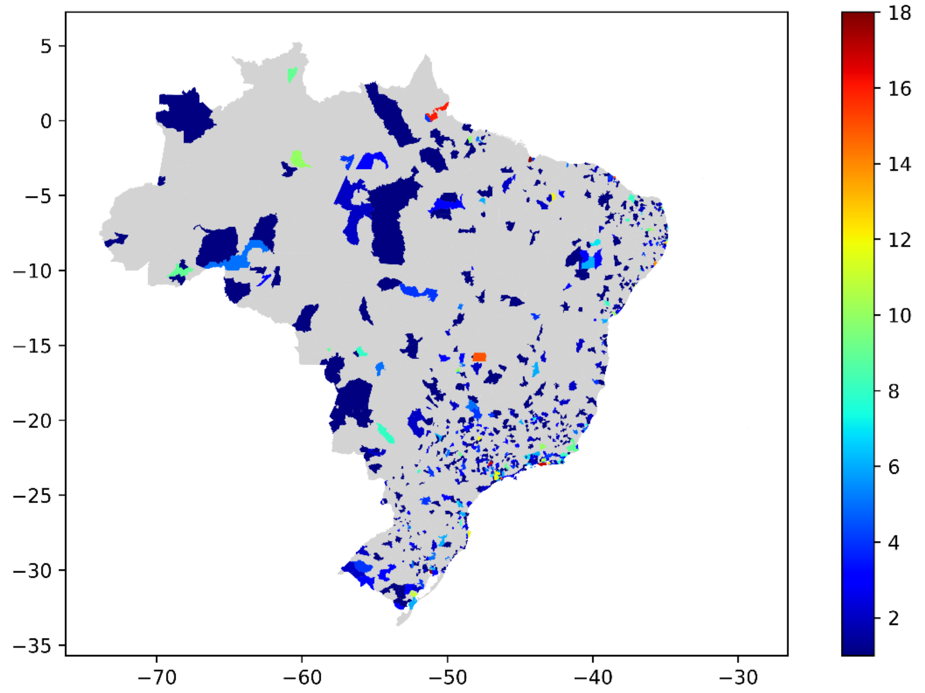

Figure S6. Topic 7 distribution on the Brazilian territory.

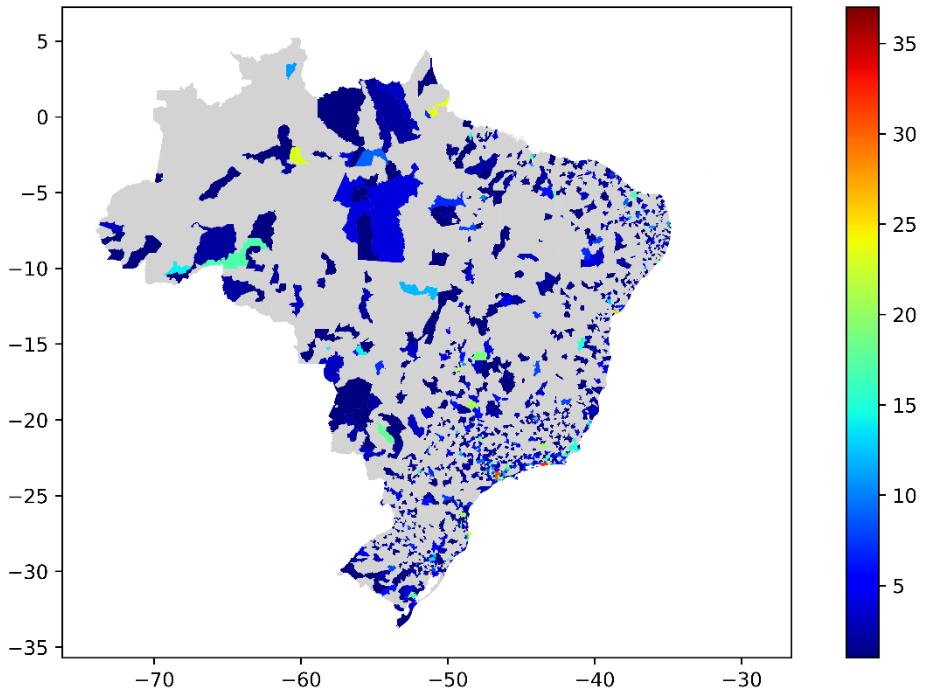

Figure S7. Topic 8 distribution on the Brazilian territory.

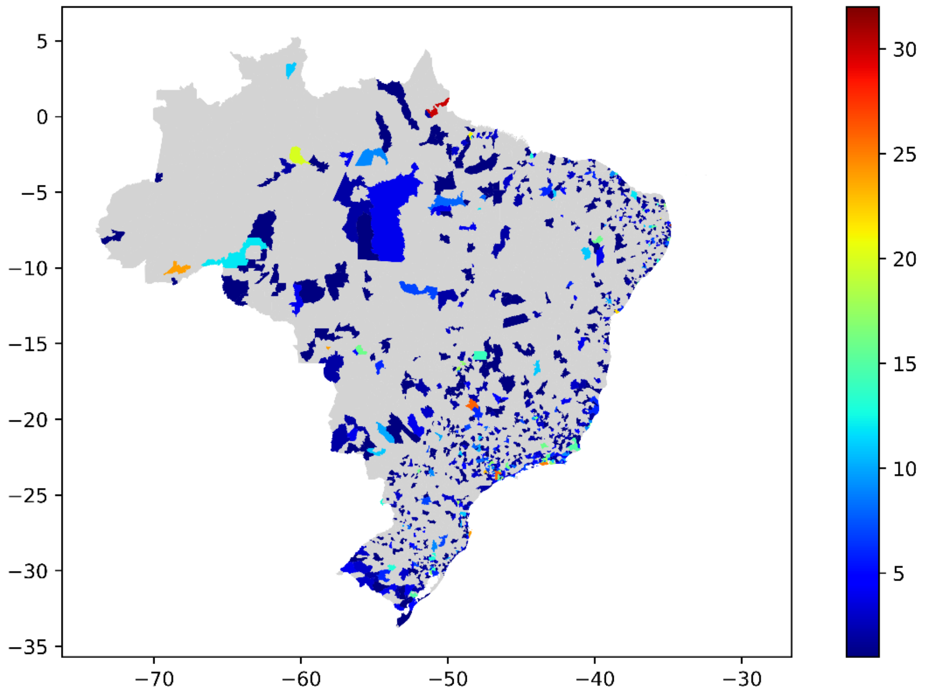

Figure S8. Topic 9 distribution on the Brazilian territory.

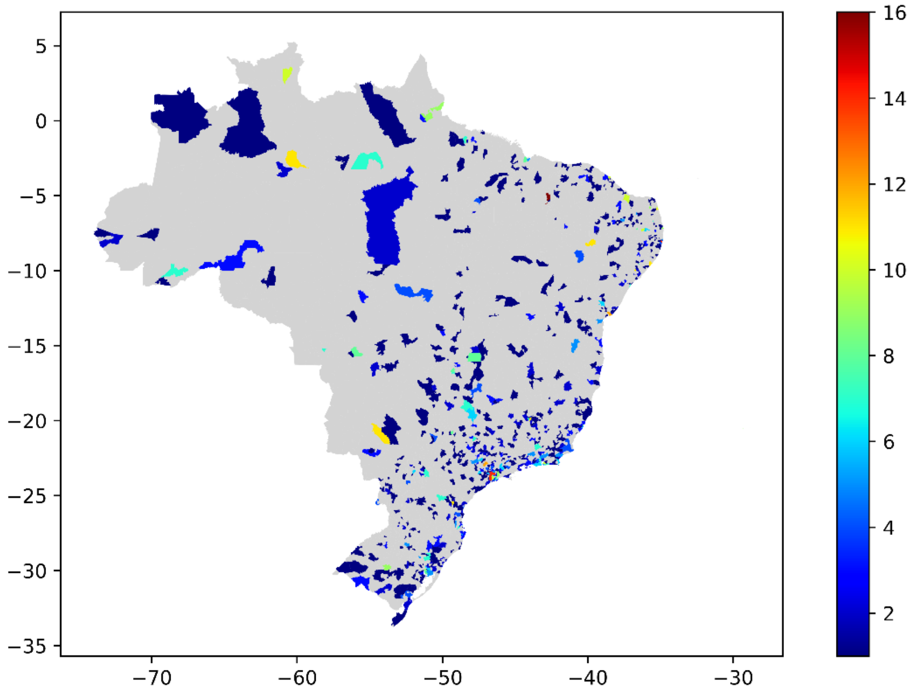

Figure S9. Topic 10 distribution on the Brazilian territory.

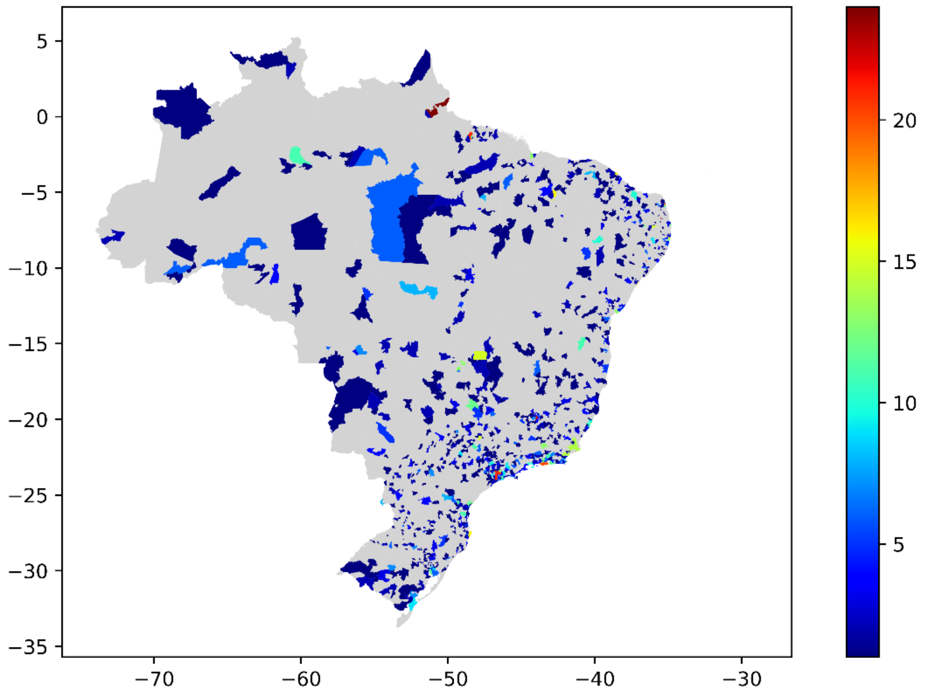

Figure S10. Topic 11 distribution on the Brazilian territory.

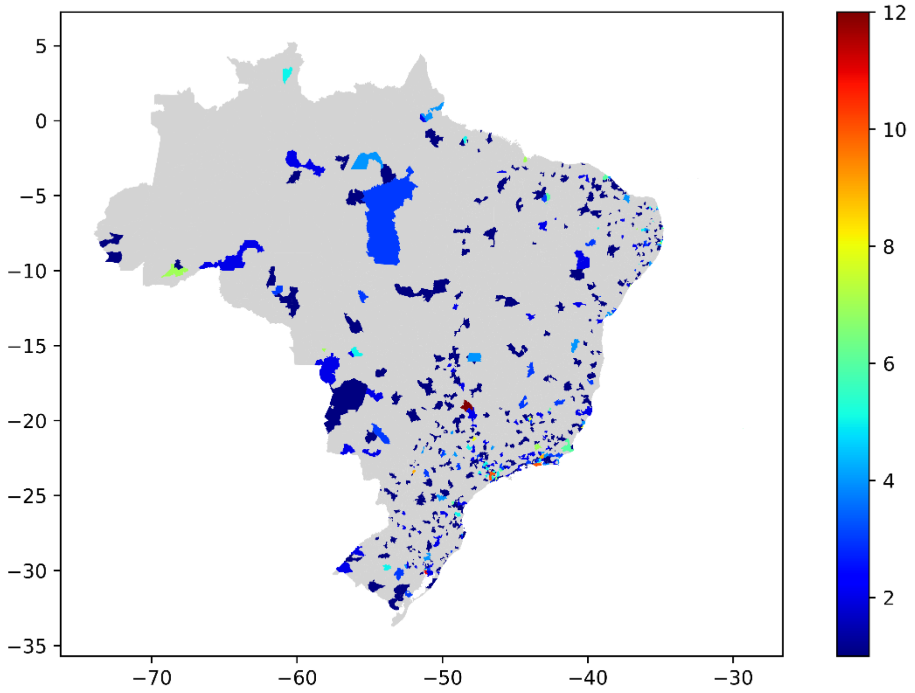

Figure S11. Topic 12 distribution on the Brazilian territory.

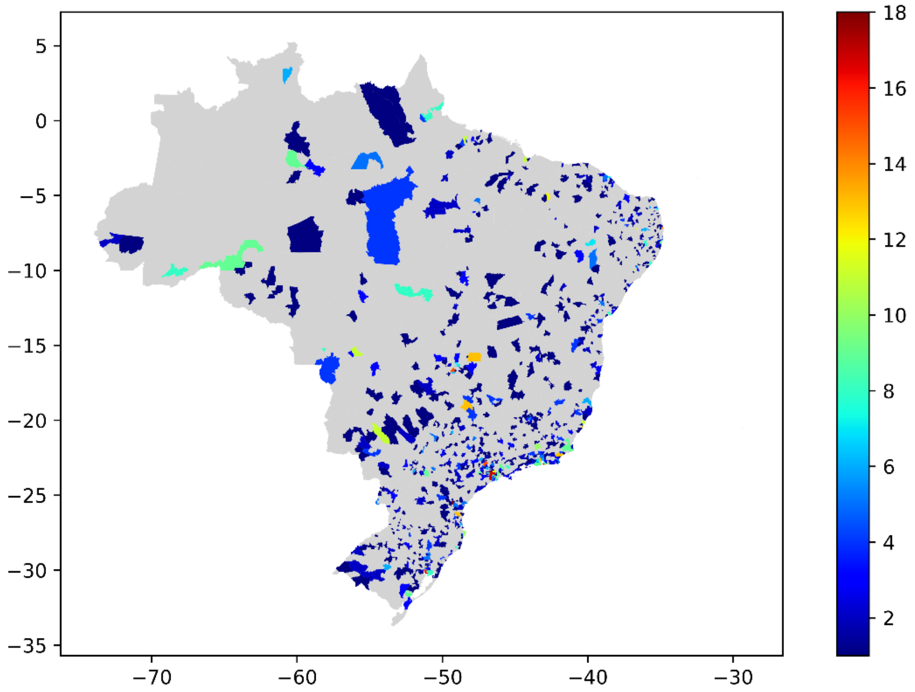

Figure S12. Topic 14 distribution on the Brazilian territory.

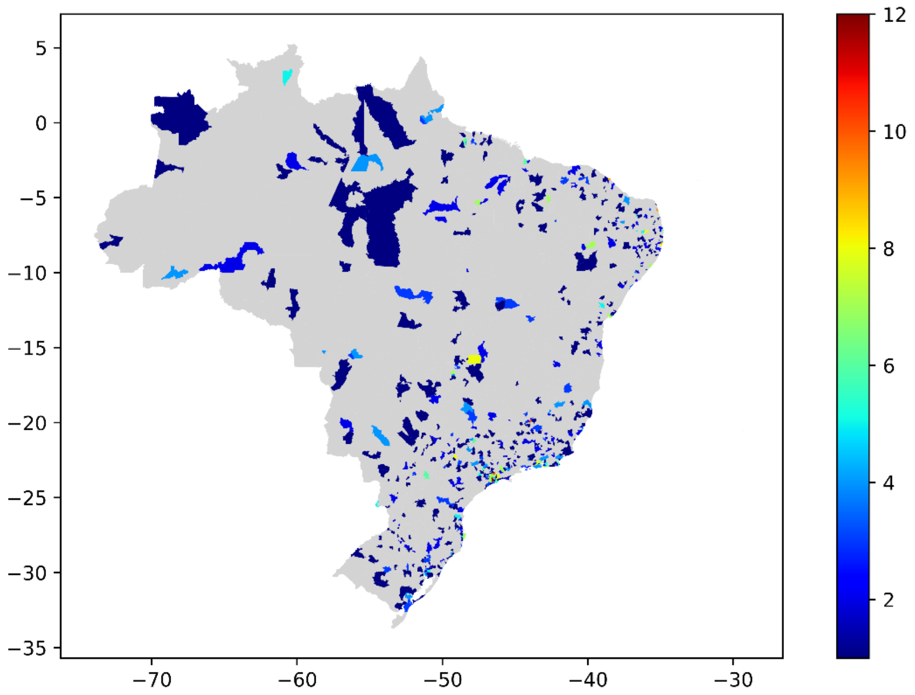

Figure S13. Topic 15 distribution on the Brazilian territory.

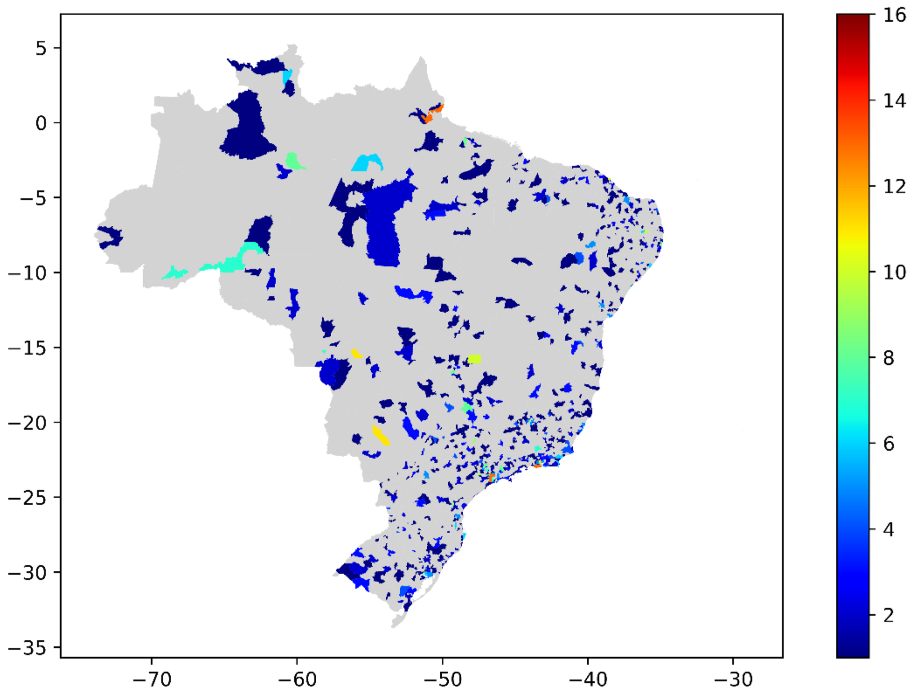

Figure S14. Topic 16 distribution on the Brazilian territory.

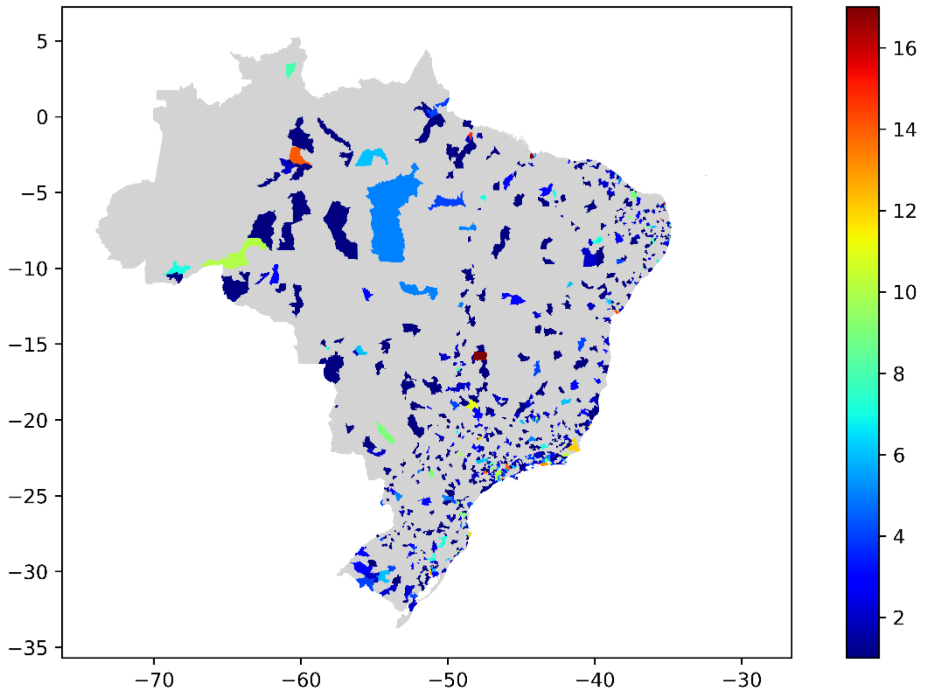

Figure S15. Topic 17 distribution on the Brazilian territory.

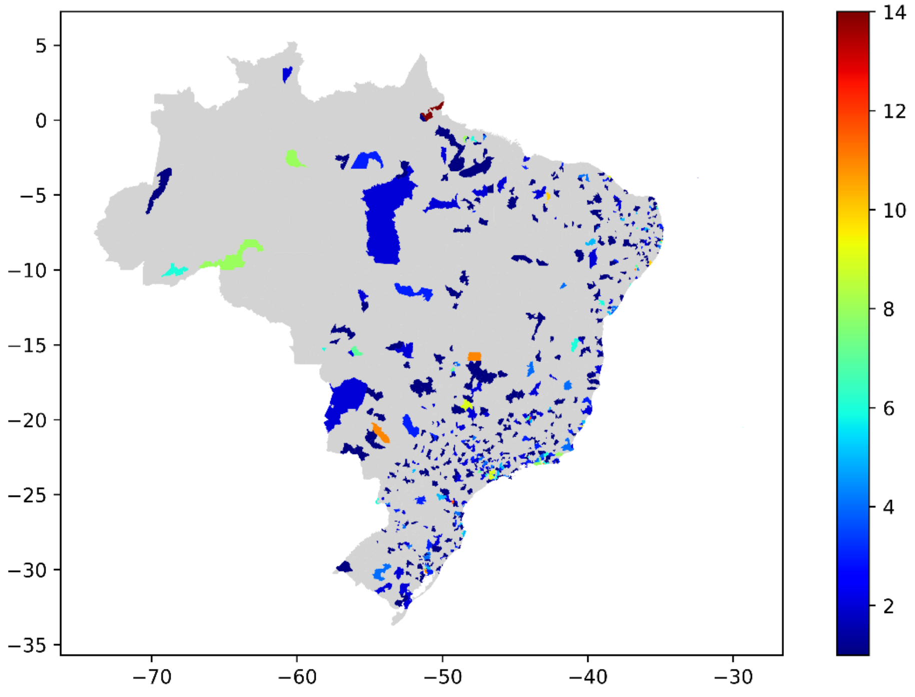

Figure S16. Topic 18 distribution on the Brazilian territory.

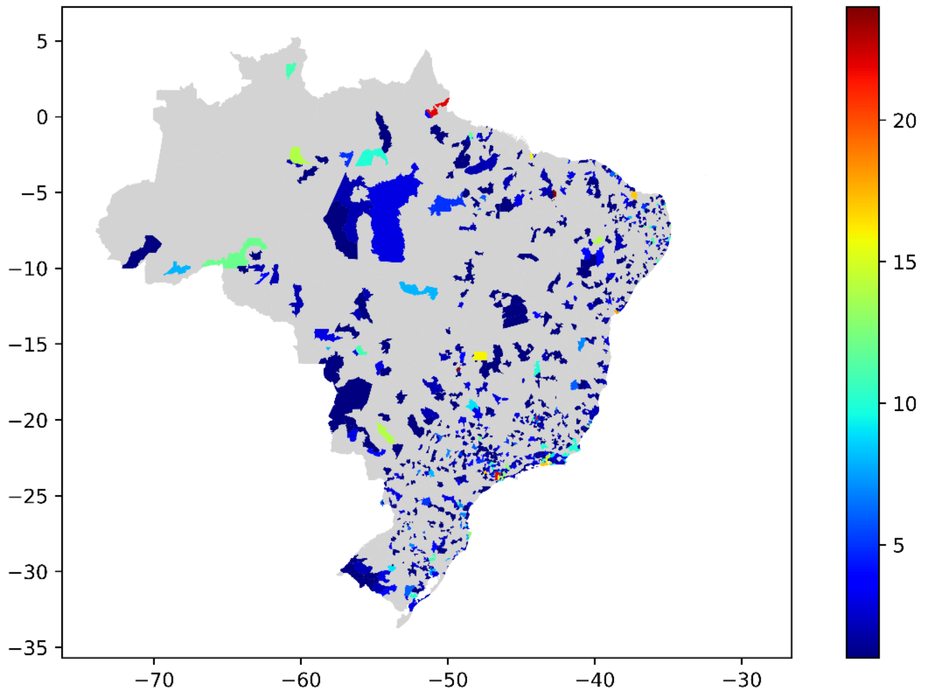

Figure S17. Topic 19 distribution on the Brazilian territory.

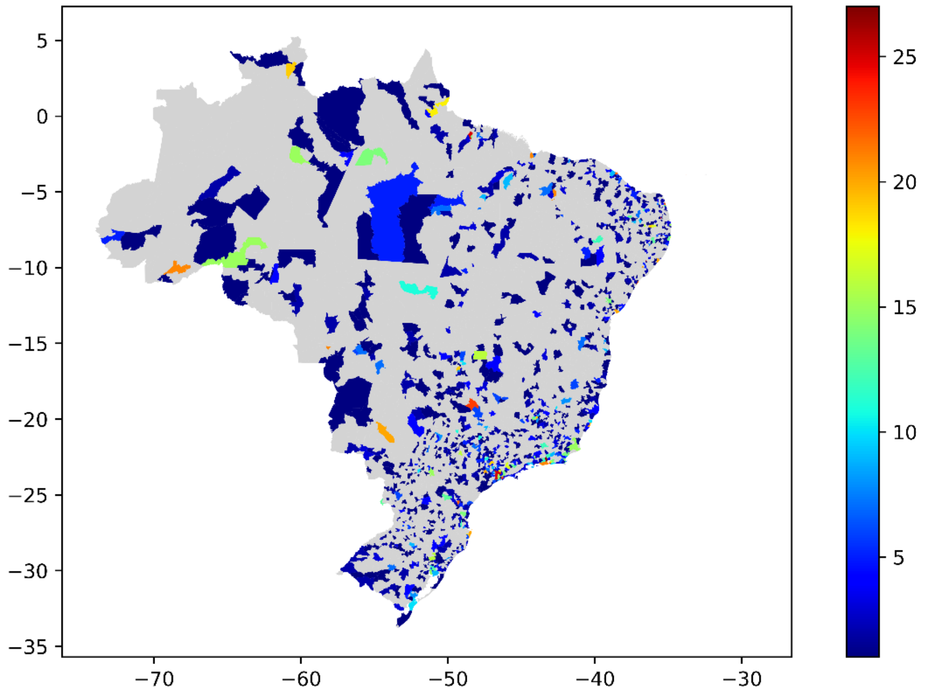

Figure S18. Topic 20 distribution on the Brazilian territory.

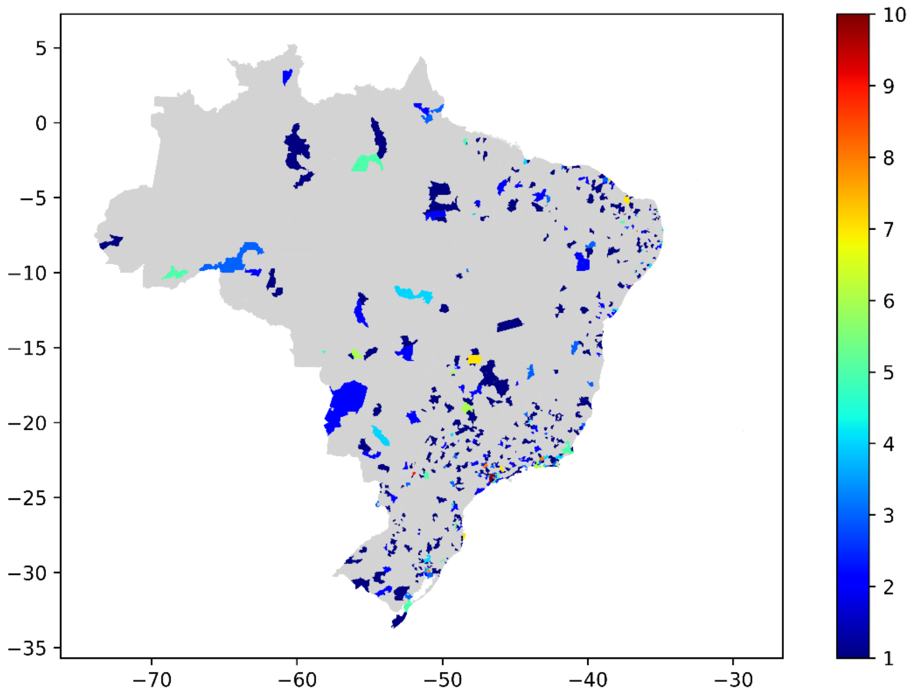

Figure S19. Topic 21 distribution on the Brazilian territory.

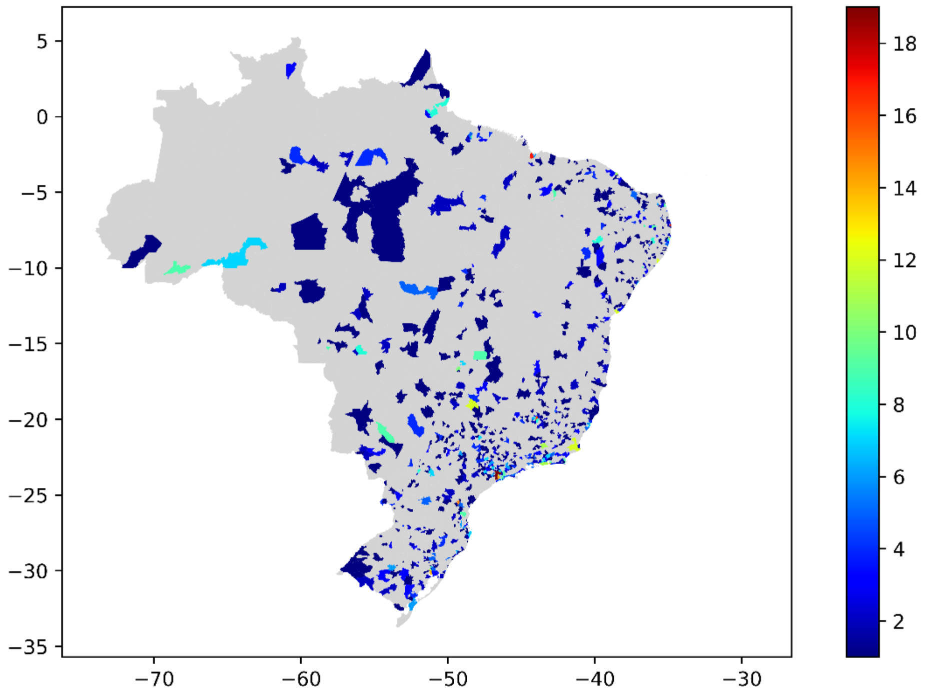

Figure S20. Topic 22 distribution on the Brazilian territory.

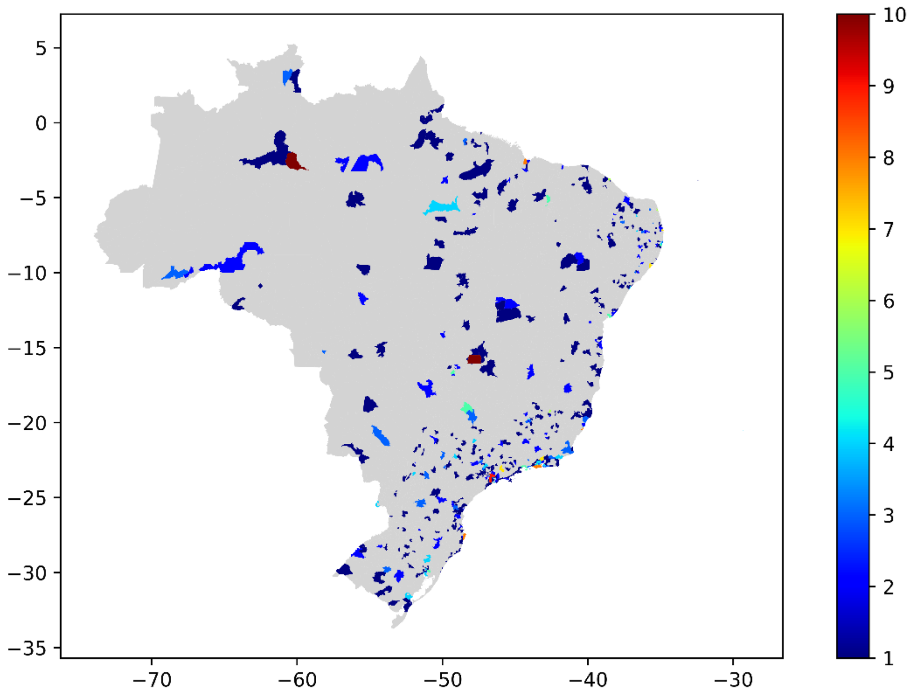

Figure S21. Topic 23 distribution on the Brazilian territory.
